# Efficacy of gamified digital health interventions for children and adolescents with autism spectrum disorder: a systematic review and meta-analysis

**DOI:** 10.1186/s13034-025-01009-w

**Published:** 2025-12-15

**Authors:** Yuxin Liu, Chi Ma, Mengmeng Zhang, Xinyi Ma, Tingxuan Liu, Feiyong Jia, Lin Du

**Affiliations:** 1 Department of Developmental and Behavioral Pediatrics, Children’s Medical Center, The First Hospital of Jilin University, Jilin University, Changchun, 130021 Jilin Province China; 2 Rehabilitation Therapeutics, School of Nursing, Jilin University, Changchun, 130021 Jilin Province China; 3 Department of Developmental and Behavioral Pediatrics, Children and Adolescents Mental Development Clinic, Children’s Medical Center, The First Hospital of Jilin University, Changchun, 130021 China

**Keywords:** Autism spectrum disorder, Gamified digital health interventions, Children and adolescents, Meta-analysis

## Abstract

**Background:**

Autism spectrum disorder is a neurodevelopmental condition with a rising prevalence and limited effective pharmacological treatments. As non-pharmacological interventions gain traction, gamified digital health interventions have emerged as a promising alternative due to their accessibility and scalability. This systematic review and meta-analysis evaluated the efficacy of gamified digital health interventions in improving key functional domains in children and adolescents with autism spectrum disorder.

**Methods:**

Following PRISMA guidelines and the Cochrane Handbook for Systematic Reviews of Interventions, a systematic search was conducted in six electronic databases (PubMed, Web of Science, EMBASE, Cochrane Library, PsycINFO, and Scopus) and reference lists of relevant articles up to November 2024. A total of 21 randomized controlled trials (RCTs) comprising 1,050 participants met the inclusion criteria. Standardized mean differences (SMDs) were pooled using a random-effects model, and subgroup analyses were conducted to explore the effects of different types of interventions.

**Results:**

Meta-analysis revealed significant improvements in emotional skills (SMD = 0.56), social skills (SMD = 0.45), executive functions (SMD = − 0.43), and motor skills (SMD = 1.53). Subgroup analyses indicated that sensor-based games demonstrated superior efficacy. However, no significant effect was observed in reducing behavioral problems (SMD = − 0.14).

**Conclusions:**

Gamified digital health interventions show promise in enhancing emotional, social, executive, and motor skills in children and adolescents with autism spectrum disorder. Future research should focus on optimizing intervention strategies, refining behavioral outcome measures, and conducting high-quality longitudinal studies to evaluate long-term effectiveness.

**Supplementary Information:**

The online version contains supplementary material available at 10.1186/s13034-025-01009-w.

## Introduction

Autism spectrum disorder (ASD) refers to a group of neurodevelopmental disorders that are present during early childhood, characterized by deficits in social interaction and communication, along with restricted and repetitive patterns of behavior, interests, or activities [1]. Globally, about 1 in 100 children are identified as having ASD [2], and these individuals often experience comorbid conditions such as emotional disorders [3], sleep disorders [4], motor abnormalities [5], and executive dysfunction [6]. ASD is a lifelong condition that continues to pose significant challenges for individuals across various domains of life as they grow older, including social interactions, academic performance, and career prospects. Their families may also face significant challenges in providing financial, emotional, and social support over the long term. The etiology of ASD involves genetic and environmental factors and is associated with alterations in multiple brain systems [7]. Its specific pathogenesis remains unclear, leading to a lack of effective treatments, and there is currently no cure for autism spectrum disorder [8]. Therefore, it is critical to implement effective interventions for autism spectrum disorder that target the reduction of its core symptoms and associated comorbidities, ultimately enhancing the quality of life for patients and alleviating the burden on their families.

Common non-pharmacological interventions for autism spectrum disorder encompass a wide range of approaches, including behavioral, educational, developmental, and therapeutic interventions. Among behavioral interventions, Applied Behavior Analysis (ABA) and Early Intensive Behavioral Intervention (EIBI) are widely used. Educational approaches often include Structured Teaching (TEACCH) and the Early Start Denver Model (ESDM) [9]. In addition to these, other frequently utilized interventions involve speech and language therapy, occupational therapy, social skills training groups, executive functioning coaching, and individualized teaching of discrete skills. These interventions have demonstrated varying degrees of success in improving communication, social interaction, and behavioral regulation in individuals with ASD. Nevertheless, they also face several limitations. Many of these interventions require specialized professional support, which may not be readily accessible in all settings. Moreover, maintaining consistency in implementation across home, school, and community environments can be challenging. Personalization remains another hurdle, as interventions must be tailored to address the diverse and evolving needs of individuals with autism spectrum disorder [10]. Recently, adjunctive and innovative approaches such as animal-assisted activities and therapies (AAAT) [11, 12], music therapy [13], and sensory integration therapy [14] have also attracted increasing attention. These therapies demonstrate unique value in improving social motivation, emotional regulation, and sensory processing in individuals with ASD by creating positive emotional experiences and providing multi-sensory stimulation. They have garnered increasing attention due to their relatively low access barriers and high emotional appeal. However, these approaches also face significant challenges, including issues with standardization and implementation safety in animal-assisted therapy, a heavy reliance on specialized therapists for music therapy, and specific physical environment requirements for sensory integration therapy. These factors constrain their accessibility, sustainability, and potential for large-scale implementation.

Digital health interventions (DHIs) go some way to making up for the shortcomings of traditional interventions. DHIs refer to health promotion, disease prevention, management, and treatment services delivered through digital technologies (e.g., mobile apps, websites, wearables, virtual reality, robotics, etc.) [15]. It offers advantages in effectiveness, accessibility, continuity, utilization, and personalization. DHIs have been shown to effectively improve mental health issues, such as depression and anxiety [16]. In addition, gamification refers to the use of game design elements and game mechanics (e.g., points, levels, rewards, challenges, etc.) in non-game settings to satisfy users’ psychological needs for competence, autonomy, and relatedness, thereby stimulating interest, motivation, and engagement [17]. Gamified digital health interventions (GDHIs), which integrate gamification elements into digital health interventions, can further boost user motivation, promote engagement in health behaviors, and improve the overall sustainability of the interventions [18]. Compared to traditional behavioral and educational interventions, GDHIs can be delivered remotely and incorporates automated elements, which reduces traditional barriers related to geography, time, and the availability of trained therapists. Furthermore, when contrasted with innovative therapies such as animal-assisted or music therapy, GDHIs not only maintain comparable levels of interactivity and emotional engagement but also demonstrate superior advantages in standardization, personalization, sustainability, and scalability. Research has shown that gamified digital health interventions can substantially alleviate mental health issues, including stress, depression, and anxiety, in adults [19]. Additionally, they have demonstrated the efficacy of these therapeutic interventions for children and adolescents afflicted with attention deficit hyperactivity disorder (ADHD) or depression [20]. This suggests that gamified digital health interventions may offer an innovative and effective treatment for autism spectrum disorder.

Although digital health interventions have been investigated in individuals with ASD, the evidence remains heterogeneous due to broad populations, varied intervention types, and inconsistent outcomes. Similarly, research on game-based digital approaches has primarily focused on mental health disorders or on selected forms such as video games, without providing a comprehensive evaluation of gamified digital health interventions specifically targeting children and adolescents with ASD [21–25]. This gap underscores the need for a systematic review and meta-analysis to clarify the efficacy of gamified digital health interventions in this population and to provide evidence-based guidance for clinical practice.

The overarching objective of this investigation was to implement a systematic review and meta-analysis of randomized controlled trials (RCTs) to evaluate the efficacy of GDHIs in children and adolescents with ASD, providing robust, evidence-based support. Specifically, this study investigated the following research questions:

1. Do GDHIs have a positive impact on emotional skills, social skills, executive functions, behavior problems, and motor skills in children and adolescents with ASD?
2. What types of GDHIs have been implemented for children and adolescents with ASD?
3. Which type of GDHIs demonstrates the most effective outcomes in improving emotional skills, social skills, executive functions, behavior problems, and motor skills in children and adolescents with ASD?

## Method

This systematic review and meta-analysis, pre-registered at PROSPERO (CRD42024622707), adhered to the Cochrane Handbook for Systematic Review of Intervention [26] and the Preferred Reporting Items for Systematic Review and Meta-Analysis (PRISMA) statement [27].

### Search strategy

A thorough search was performed across six databases(PubMed, Web of Science, EMBASE, Cochrane Library, PsycINFO, and Scopus) from the library’s inception until November 29, 2024. We only restricted participants and interventions to maintain the comprehensiveness of the search. The keywords such as “Autism Spectrum Disorder”, “Video Games”, and “game” were combined and searched across the six databases using free-text words, Medical Subject Headings (MeSH), and Boolean calculations. The complete Search strategy and database results are detailed in the Supplementary File 1. In addition, reference lists from related systematic reviews and meta-analyses were manually searched to identify and retrieve other pertinent studies.

### Inclusion and exclusion criteria

The inclusion criteria are built on the participants, intervention, comparison, outcome, and study design (PICOS) framework.

1. Participants: Participants included in the studies were children and adolescents (≤ 18 years) with a confirmed diagnosis of ASD, diagnosed using standardized tools (e.g., DSM-IV and DSM-5, ICD-10, and ADOS-2) or other standardized diagnostic criteria.
2. Intervention: Gamified digital health interventions, defined broadly as digital programs incorporating game-based or game-like elements (e.g., serious games, virtual reality, neurofeedback games, computer-assisted programs with rewards or avatars, or robot-assisted interventions with gamified features). No restriction was applied regarding frequency or duration.
3. Comparison: The control group was treated with usual care, placebo, or waiting list control.
4. Outcomes: Outcomes of interest included improvement in any relevant functions (e.g., emotional skills, social skills, executive functions, behavioral problems, motor skills).
5. Study design: The study design was implemented using a randomized controlled trial model, with all selected trials published in English-language journals.

The exclusion criteria were as follows:

1. Duplicate publications;
2. Studies without an accessible full text;
3. Non-randomized studies, conference abstracts, or reviews;
4. Interventions where gamification was not a meaningful component of the digital program.
5. Studies with mixed populations (e.g., ASD with other neurodevelopmental disorders), unless ASD-specific data could be extracted;

### Study selection

The database results were imported into EndNote 21 for literature management, and duplicates were removed. Two independent reviewers assessed the titles and abstracts of the remaining records according to the eligibility criteria. The final inclusion of articles that met the established criteria for this systematic review and meta-analysis was determined following a thorough screening of the full texts. Any disagreements and discrepancies were resolved via consensus-based mediation by a third reviewer.

### Quality assessment

To evaluate the risk of bias, we utilized the Cochrane Tool for Risk of Bias (RoB 2, Version 2) as outlined in the Cochrane Handbook. This assessment was carried out in five key aspects: randomization process, deviations from intended interventions, missing outcome data, outcome measurement, and selection of reported result [28]. Each aspect was systematically evaluated and categorized as “low,” “some concerns,” or “high” to determine overall risk-of-bias judgments.

The quality of evidence was systematically evaluated by means of the GRADEpro GDT software, guided by the Grading of Recommendations, Assessment, Development, and Evaluation (GRADE) methodology [29]. This approach categorizes evidence into four distinct levels: “high”, “moderate”, “low”, or “very low”, which is predicated on a systematic assessment of five key domains, namely the risk of bias, inconsistency, indirectness, imprecision, and publication bias.

### Data extraction

In accordance with the guidelines from the Cochrane Handbook for Systematic Reviews of Interventions, a customized data extraction form was created. This form encompassed the following essential elements: study details (author, year, country), participant characteristics (diagnostic criteria, age, sex, sample size), intervention details (intervention form, delivery modality, duration), and control group. To calculate effect sizes, the mean (SD) of outcome measures post-intervention was collected for each study. For any outcome where the effect size of interest was not explicitly reported, the available data were analyzed using the approach recommended in the Cochrane Handbook for Systematic Reviews of Interventions [26]. When outcome data were incomplete or unavailable, we attempted to contact the study authors to request additional information.

### Data analysis

This review used Review Manager (RevMan) version 5.3 for data pooling in the meta-analysis. For data that could not be aggregated, a qualitative systematic review was used. For continuous outcomes, the aggregated results were expressed as standardized mean differences (SMDs) along with 95% confidence intervals (CIs) to account for heterogeneity in measurement instruments across studies. Summary estimates for all outcome measures were generated using a random-effects model, accommodating variations in both intervention characteristics and outcome measures [30]. The magnitude of the effect sizes was evaluated according to the criteria established by Cohen: an SMD of 0.2 was considered indicative of a small effect, 0.5 of a medium effect, and 0.8 of a large effect [31]. The statistical significance of the overall effect was established through the application of a p-value cut-off of 0.05.

The I² statistic was used to quantify and illustrate the extent of statistical heterogeneity, categorized as unimportant (0–40%), moderate (30–60%), substantial (50–90%), or considerable (75–100%), in accordance with the Cochrane Handbook for Systematic Reviews of Interventions [26]. Subgroup analyses were performed based on intervention type to explore the efficacy of GDHIs with different delivery modalities. Sensitivity analyses were performed to examine how individual studies influenced the overall effect size and to pinpoint studies that may have introduced significant heterogeneity. These studies were then excluded to assess changes in the pooled results. Publication bias was not investigated because no more than 10 studies were included in each analysis.

## Result

### Literature search

**Fig. 1:**
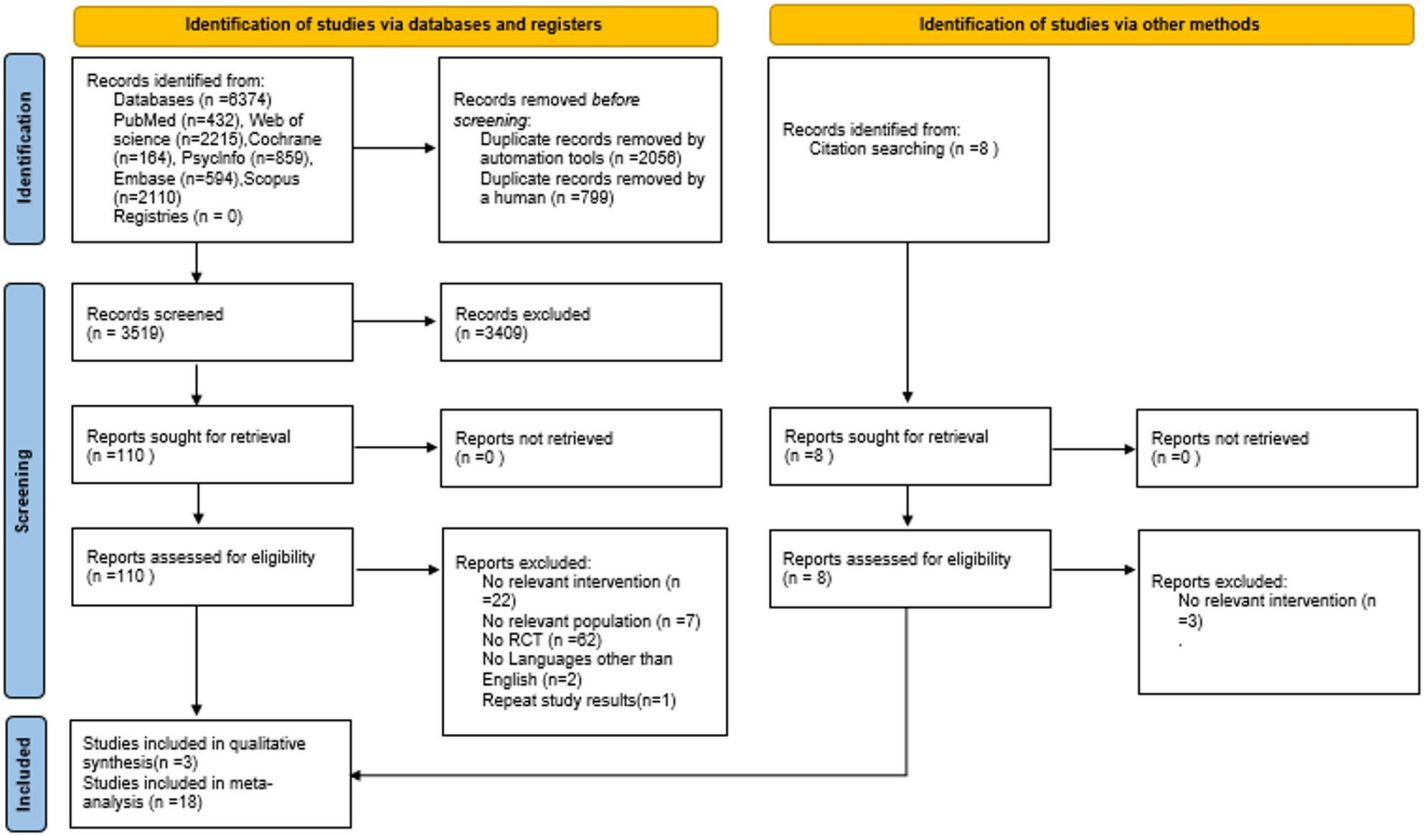
PRISMA flowchart of study search and selection strategy

Figure 1 depicts the search results and the literature selection process. We identified 6374 records from four databases, and 8 additional records were identified from searching the reference lists of relevant systematic reviews and meta-analyses. After eliminating 2855 duplicate records, the remaining 3527 article titles and abstracts were subjected to screening, leading to the exclusion of 3409 articles. Of the 118 full-text articles that were assessed for eligibility, with 21 studies [32–52] included in the systematic review. Among these,18 studies [32–38, 40, 42–44, 46–52] were deemed appropriate for meta-analysis as they reported sufficient data.

### Study characteristics

The key characteristics of the included studies are outlined in Table 1. The analysis encompassed 21 studies, all of which were randomized controlled trials (RCTs) published from 2011 to 2024. These studies were conducted in Europe (*n* = 8) [33, 35, 36, 39, 48–51], Asia (*n* = 5) [36, 42, 44, 46, 52], North America (*n* = 7) [34, 37, 38, 40, 41, 43, 47], Oceania (*n* = 1) [32]and South America (*n* = 1) [45].

#### Participant characteristics

A total of 1,050 participants, consisting of children and adolescents aged 1–18 years diagnosed with ASD, fulfilled the inclusion criteria across the included studies. The proportion of females in the total sample was approximately 14.99%, except for four studies [33, 41, 45, 46] that did not report sex ratios. In 16 studies [32–40, 44, 46, 48–52], participants were diagnosed with ASD by licensed medical professionals based on standardized diagnostic criteria such as DSM-IV, DSM-5, ICD-10, or assessment tools like ADOS-2. One study [47] reported diagnoses made by a licensed medical professional without specifying the diagnostic tools used. For the remaining studies [41–43, 45], although explicit diagnostic criteria were not detailed, it is presumed that participants were similarly diagnosed according to recognized standardized protocols.

#### Interventions and controls

The mean intervention range was 4 weeks to 20 weeks (M = 8.63, SD = 3.40). The gamified digital health interventions in the study were classified into four categories based on delivery modality: computer game [32–40, 43, 47, 49, 51], robot [48], sensor game [42, 44, 46, 50, 52], and video game [41, 45].

### Risk-of-bias assessment

**Fig. 2:**
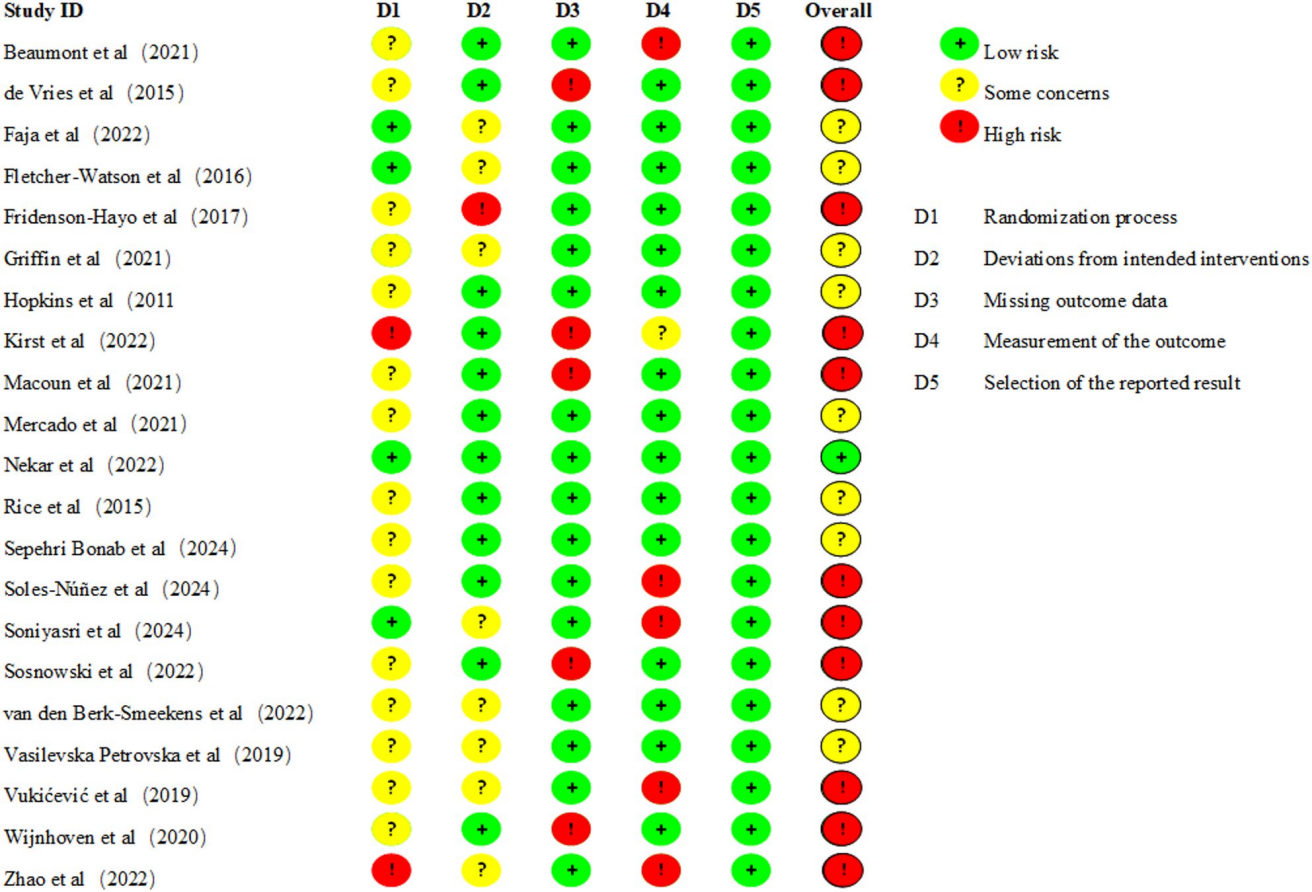
Risk of bias for all included studies

**Fig. 3:**
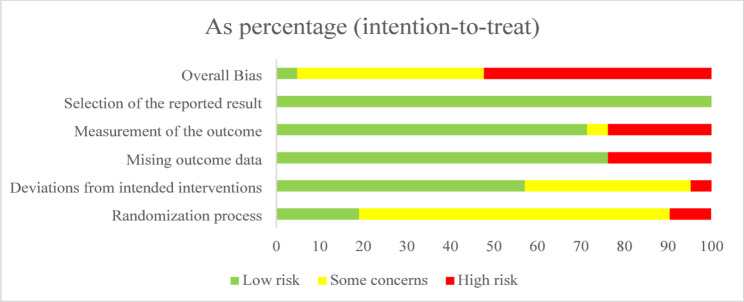
Summary of distribution of different biases

Figures 2 and 3 present the updated risk of bias assessments for the 21 included randomized controlled trials. Among these studies, one [42] was rated as “low risk”, nine [34, 35, 37, 38, 41, 43, 44, 48, 49] as “some concerns”, and eleven [32, 33, 36, 39, 40, 45–47, 50–52] as “high risk”as. The most affected domains were “bias arising from the randomization process”, “bias due to missing outcome data”, and “bias in measurement of the outcome”, reflecting common methodological challenges in gamified digital health intervention studies. Detailed domain-level judgments are available in Supplementary File 2, and a comparison of original and revised overall judgments is provided in Supplementary File 3.

### Efficacy of gamified digital health interventions for children and adolescents with autism spectrum disorder

A comprehensive analysis of the results from 21 studies revealed that gamified digital health interventions significantly enhance emotional skills, social skills, executive functions, and motor skills in children and adolescents with ASD. However, the impact on reducing problem behaviors was found to be non-significant.

#### Emotional skills

**Fig. 4:**
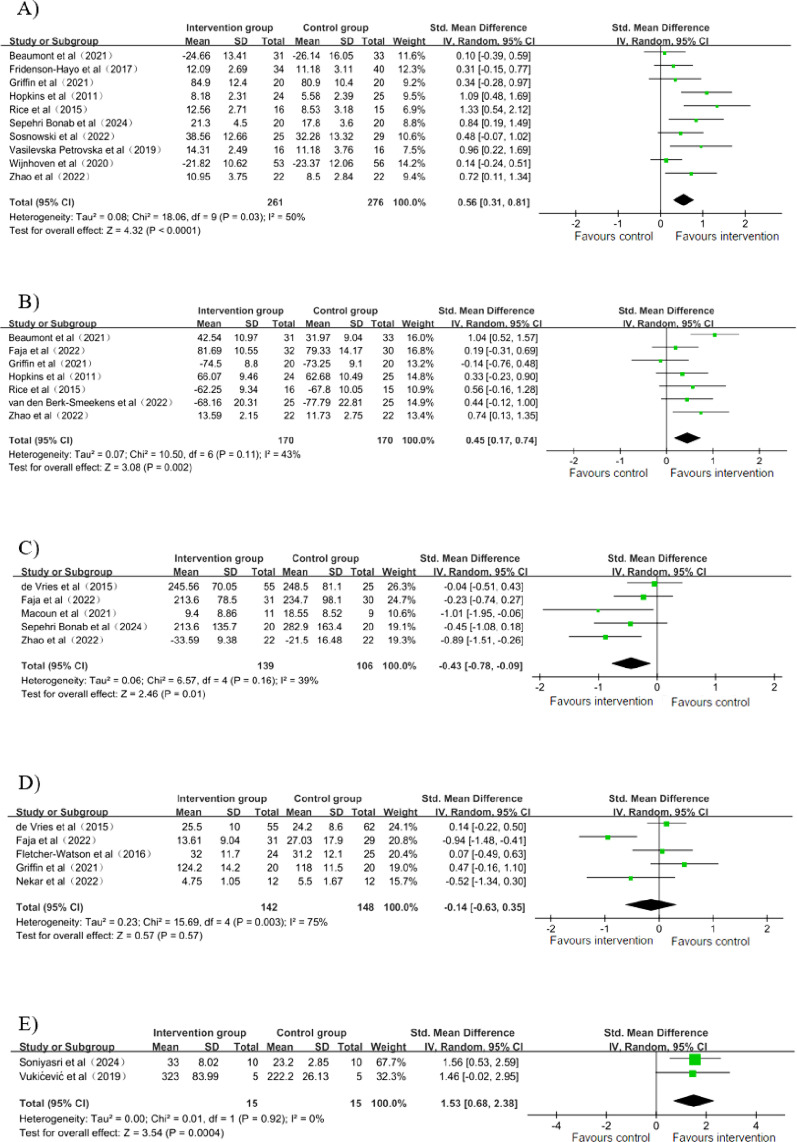
Forest plot for meta-analysis regarding the effect of gamified digital health interventions. **a** Forest plot for meta-analysis regarding the effect of gamified digital health interventions on emotional skills. **b** Forest plot for meta-analysis regarding the effect of gamified digital health interventions on social skills. **c** Forest plot for meta-analysis regarding the effect of gamified digital health interventions on executive functions. **d** Forest plot for meta-analysis regarding the effect of gamified digital health interventions on behavior problems. **e** Forest plot for meta-analysis regarding the effect of gamified digital health interventions on motor skills

11 studies [32, 36–39, 43, 44, 47, 49, 51, 52] assessed the impact of the interventions on emotional skills. Among the 11 studies, 10 studies [32, 36–38, 43, 44, 47, 49, 51, 52] containing data from 537 participants were identified as suitable for meta-analysis. The meta-analysis indicated that GDHIs exhibited a substantial enhancement in the emotional skills of children and adolescents diagnosed with ASD, in comparison with the control group, indicating a medium effect size (SMD = 0.56; 95% CI: 0.31 to 0.81; *p* < 0.0001, Fig. 4a). Heterogeneity was moderate (I² = 50%, *p* = 0.03). After excluding one study [43], heterogeneity decreased to 40%, and the effect size was reduced to 0.49 (SMD = 0.49; 95% CI: 0.25 to 0.73; *p* < 0.0001). This decrease could be attributed to the excluded study’s intervention, which was specifically designed to enhance emotional skills through targeted training in eye gaze, joint attention, and facial recognition. In addition, one study [39] that lacked available data showed that after a six-week intervention with Zirkus Empathico, there were significant improvements in both empathy (medium effect size: d = 0.71) and emotion recognition (medium effect size: d = 0.50) in the intervention group compared with the control group.

#### Social skills

Eight studies [32, 34, 37, 38, 43, 45, 48, 52] assessed the impact of the interventions on social skills. Seven studies [32, 34, 37, 38, 43, 48, 52] (*n* = 340 participants) were included in the meta-analysis on social skills. The meta-analysis revealed that compared with the control group, GDHIs exhibited a significant positive effect on social skills in children and adolescents with ASD (SMD = 0.45; 95% CI: 0.17 to 0.74; *p* = 0.002; I² = 43%, Fig. 4b), indicating a moderate effect size. This heterogeneity disappeared (I² = 0%) following the exclusion of a single study [32], resulting in a pooled SMD of 0.34 (95% CI: 0.31 to 0.81; *p* = 0.006). This may be ascribed to the fact that the gamified digital intervention in this study, which primarily focused on social skills, significantly enhanced participants’ social skills. One study [45], for which no usable data were available, found that communication skills showed significant improvement following the video game intervention, with an average increase of 27.8% compared with the control group.

#### Executive functions

Six studies [33, 34, 40, 41, 44, 52] evaluated the effects of the interventions on executive functions. A total of five studies [33, 34, 40, 44, 52] (involving 245 participants) were incorporated into the meta-analysis for the purpose of investigating the effects of GDHIs on executive functions. The analysis yielded a pooled result that indicated significant effects of these interventions (SMD = − 0.43; 95% CI: − 0.78 to − 0.09; *p* = 0.01; I^2^ = 39%, Fig. 4c). One study [41] which was not incorporated into the meta-analysis due to unavailable data suggested that Farmer Keeper, a BCI video game, significantly improved the attention-related executive functions in children with autism. We attempted to contact the study authors to obtain the missing data, but no additional information was available.

#### Behavior problems

The meta-analysis of five studies [33–35, 37, 42] involving 290 participants demonstrated that there was no substantial decrease in behavioral problems within the intervention group compared to the control group (SMD: − 0.14; 95% CI: − 0.63 to 0.35; *p* = 0.57; I^2^ = 75%, Fig. 4d). Following the exclusion of a single study [34], heterogeneity decreased to 16%, and the effect size reduced to 0.11 (SMD = 0.11; 95% CI: − 0.19 to 0.40; *p* = 0.48). This decline may be ascribed to the fact that the control group in this study was a waitlist control, which differed from the control groups in the other studies.

#### Motor skills

Only two studies [46, 50](*n* = 30) investigated the influence of GDHIs on motor skills. The pooled SMD revealed that GDHIs had a significant effect on motor skills, suggesting a considerable effect size (SMD: 1.53; 95% CI: 0.68 to 2.38; *p* = 0.00004; I^2^ = 0%, Fig. 4e).

#### Subgroup analyses

Subgroup analyses were conducted according to various types of interventions, with detailed results presented in Table 2. The results indicated that sensor games resulted in greater improvements in emotional skills, social skills, executive functions, and behavioral problems compared with computer games.

#### Summary of outcome measures and informants

Beyond differences in intervention delivery modalities, the studies included in the meta-analysis also varied in terms of outcome assessment methods. Emotional outcomes were measured using both informant-based questionnaires (e.g., SCAS, ERC) and child performance tasks (e.g., Face task, NEPSY-II). Social skills were primarily assessed via parent- or teacher-reported scales (e.g., SSRS, SRS-2), with some clinician-rated instruments (e.g., PEP-3). Executive functions were mostly evaluated through performance-based tasks (e.g., SSRT, Flanker). Behavioral problems relied largely on parent questionnaires (e.g., DBDRS, RBS-R), complemented by observational coding (e.g., BOSCC). Motor skills were assessed exclusively with standardized examiner-rated tools (e.g., TGMD-2, DASH-2). Notably, subjective domains such as emotional and behavioral functioning are particularly susceptible to informant bias, as discrepancies between self- and proxy-reports have been consistently documented in pediatric populations [53, 54]. A detailed mapping of outcome domains, instruments, informants, and classification into subjective versus objective assessments is presented in Supplementary File 4.

#### Sensitivity analysis

The research findings on emotional skills, social skills, executive functions, and behavioral problems showed a degree of heterogeneity. This heterogeneity was reduced by sequentially removing individual studies. The effect sizes remained largely unchanged after removing each study one by one from the forest plot, indicating the robustness and reliability of the results.

#### Quality of evidence

Following the GRADE methodology guidelines, the certainty of evidence for emotional skills was rated as “moderate”, downgraded one level due to risk of bias, mainly reflecting concerns in the domains of randomization process and deviations from intended interventions. The certainty of evidence for behavior problems was rated as very low, downgraded for risk of bias, inconsistency (substantial heterogeneity), and very serious imprecision due to small sample size and wide confidence intervals crossing the line of no effect. The certainty of evidence for social skills, executive functions, and motor skills was rated as low, primarily due to risk of bias and imprecision. The details of the assessment and results for each outcome are presented in Table 3.

**Table 1:** Characteristics of the included studies

Study	Country	Participant characteristics				Intervention	Delivery modality	Control group	Duration
		Diagnostic criteria	Age (year)	Sex, Male/female	Sample size				
Beaumont et al. (2021)	Australia	DSM-5	7–12	60/10	70	SAS	Computer	Placebo digital intervention	10 weeks
de Vries et al. (2015)	Netherlands	DSM-IV	8–12	NR	90	Brain game	Computer	Placebo digital intervention	6 weeks
Faja et al. (2022)	USA	DSM-5	7–11	63/7	70	Computer games and metacognition coaching	Computer	Waitlist	10 weeks
Fletcher-Watson et al. (2016)	UK	ADOS-2	1–6	43/11	54	Find Me	Computer	TAU	2 months
Fridenson-Hayo et al. (2017)	Israel and Sweden	DSM- IV, DSM-5 ,ICD-10	6–9	66/8	74	Emotiplay	Computer	Waitlist	8 weeks
Griffin et al. (2021)	USA	ADOS-2	10–18	33/7	40	SAGA	Computer	TAU	10 weeks
Hopkins et al. (2011)	USA	DSM-IV	6–15	44/5	49	FaceSay	Computer	Placebo digital intervention	6 weeks
Kirst et al. (2022)	Germany and Austria	ICD-10	5–10	69/13	82	Zirkus Empathico (ZE)	Computer	Placebo digital intervention	6 weeks
Macoun et al. (2021)	Canada	DSM-IV	6–12	17/3	20	Caribbean Quest	Computer	Waitlist	8 weeks
Mercado et al. (2021)	Mexico	NR	4–13	NR	26	Farmer Keeper	Video game console	Placebo digital intervention	10 weeks
Nekar et al. (2022)	Korea	NR	6–18	22/2	24	AR game	Sensor game console	TAU	4 weeks
Rice et al. (2015)	USA	NR	5–11	28/3	31	FaceSay	Computer	Placebo digital intervention	10 weeks
Sepehri Bonab et al. (2024)	Iran	DSM-5	7–10	40/0	40	Xbox 360 Kinect Game	Sensor game console	Placebo digital intervention	8 weeks
Soles-Núñez et al. (2024)	Perú	NR	NR	NR	60	Video game developed with the Unity 3D	Video game console	TAU	2 months
Soniyasri et al. (2024)	India	DSM-5	5–10	NR	20	Kinect Xbox games	Sensor game console	TAU	8 weeks
Sosnowski et al. (2022)	USA	Medical professional	4–14	47/7	54	Lookware	Computer	Placebo digital intervention	6 weeks
van den Berk-Smeekens et al. (2022)	Netherlands	DSM- IV	3–8	42/8	50	Robot	Robot	TAU	20 weeks
Vasilevska Petrovska et al. (2019)	North Macedonia	ICD-10	7–15	23/10	33	Ucime Emocii	Computer	TAU	6 weeks
Vukićević et al. (2019)	Serbia	DSM-5	9–13	10/0	10	Fruits	Sensor game console	TAU	5 weeks
Wijnhoven et al. (2020)	Netherlands	DSM- IV	8–16	84/25	109	Mindlight	Computer	Placebo digital intervention	6 weeks
Zhao et al. (2022)	China	DSM-5	3–6	35/9	44	VR technology	Sensor game console	TAU	12 weeks

*NR* not reported, *ADOS-2* Autism Diagnostic Observation Schedule-Second Edition, *DSM-4 IV and-5* Diagnostic and Statistical Manual of Mental Disorders, 4th edition and 5th edition, *ICD-10* International Classification of Diseases, 10th Revision

**Table 2:** Subgroup analysis categorized by the type of gamified digital health intervention

Outcomes	Subgroup (delivery methods of interventions)	No. of studies	No. of participants	SMD	Pooled effect size [95%CI]	*P*-value for pooled result	I^2^ (%)
Emotional skills	Total	10	537	0.56	[0.31, 0.81]	< 0.0001	50
	Computer	7	399	0.54	[0.19, 0.88]	0.002	63
	Sensor game console	3	138	0.66	[0.31, 1.00]	0.0002	0
Social skills	Total	7	340	0.45	[0.17, 0.74]	0.002	43
	Computer	5	246	0.40	[0.00, 0.80]	0.05	58
	Sensor game console	1	44	0.74	[0.13, 1.35]	0.02	
	Robot	1	50	0.44	[− 0.12, 1.00]	0.13	
Executive functions	Total	5	245	− 0.43	[− 0.78, − 0.09]	0.01	39
	Computer	3	161	− 0.28	[− 0.71, 0.15]	0.20	38
	Sensor game console	2	84	− 0.67	[− 1.11, − 0.23]	0.003	0
Behavior problems	Total	5	290	− 0.14	[− 0.63, 0.35]	0.57	75
	Computer	4	266	− 0.07	[− 0.63, 0.49]	0.80	79
	Sensor game console	1	24	− 0.52	[− 1.34, 0.30]	0.21	

**Table 3:** Grade evidence profile

Certainty assessment							No. of patients		Effect		Certainty
No. of studies	Study design	Risk of bias	Inconsistency	Indirectness	Imprecision	Other considerations	Game-based digital health interventions	Placebo digital intervention, waitlist or TAU	Relative (95% CI)	Absolute (95% CI)	
*Emotional skills*											
10	Randomised trials	Serious^a^	Not serious	Not serious	Not serious	None	261	276	–	SMD 0.56 higher (0.31 higher to 0.81 higher)	⨁⨁⨁◯ Moderate^a^
*Social skills*											
7	Randomised trials	Serious^a^	Not serious	Not serious	Serious^b^	None	170	170	–	SMD 0.45 higher (0.17 higher to 0.74 higher)	⨁⨁◯◯ Low^a, b^
*Executive functions*											
5	Randomised trials	Serious^a^	Not serious	Not serious	Serious^b^	None	139	106	–	SMD 0.43 lower (0.78 lower to 0.09 lower)	⨁⨁◯◯ Low^a, b^
*Behavior problems*											
5	Randomised trials	Serious^a^	Serious^c^	Not serious	Very serious^d^	None	142	148	–	SMD 0.14 lower (0.63 lower to 0.35 higher)	⨁◯◯◯ Very low^a, c,d^
*Motor skills*											
2	Randomised trials	Serious^a^	Not serious	Not serious	Serious^b^	None	15	15	–	SMD 1.53 higher (0.68 higher to 2.38 higher)	⨁⨁◯◯ Low^a, b^

*CI* confidence interval, *SMD* standardised mean difference

^a^Downgraded one level for risk of bias: most studies had ≥ 1 domain rated as “some concerns” or “high risk”

^b^Downgraded one level for imprecision: total sample < 400 participants

^c^Downgraded one level for inconsistency: substantial heterogeneity (I² >50%)

^d^Downgraded two levels for very serious imprecision: total sample < 400 and 95% CI crossed the line of no effect

## Discussion

To the best of our knowledge, this represents the first systematic review and meta-analysis of randomized controlled trials focusing solely on the efficacy of GDHIs for children and adolescents with ASD. Novel findings from 21 studies involving 1050 participants revealed that these interventions primarily included computer games, video games, sensor games, and robots. The interventions showed potential in improving several key skills (e.g., emotional skills, social skills, executive functions, and motor skills)in children and adolescents with ASD, although no significant effects were observed for reducing problem behaviors.

Results from 11 studies suggested improvements in emotional skills, which may be attributed to the ability of these interventions to teach participants to recognize multiple emotional cues, such as facial expressions, vocal intonation, and body language, through interactive tasks [32, 36, 39, 49]. Additionally, they provide practical emotion regulation techniques, including deep breathing and mindfulness [32, 39]. This dual mechanism of action underscores the potential of GDHIs as a powerful tool for improving emotional skills in this population. This finding aligns with the conclusions of Xu et al. [22], who discovered that digital interventions significantly improved the emotional skills of children and adolescents with ASD. It also demonstrated the findings of Bryant et al. [20] that gamified digital health could ameliorate depression in children and adolescents.

GDHIs demonstrate significant efficacy in enhancing social skills among children and adolescents with ASD. According to social motivation theory [55], the social deficits observed in individuals with ASD are partly due to reduced social motivation, which hinders their ability to attend to and learn from socially relevant information in their environment. This lack of motivation contributes to deficits in social skills and impaired interactions with others. From this theoretical perspective, GDHIs stimulate children’s interest and motivation in social interactions by simulating virtual scenes of social interactions, helping them to learn social behaviors and practice communication skills in a less anxious and more rewarding environment. The results are in good consistent with Wang et al. [21] and Azadboni et al. [25], who reported that digital interventions enhance social skills.

Positive effects on executive functions were also observed. Executive functions are a series of higher-order cognitive abilities that can help individuals effectively plan, organize, control behavior, solve problems, and achieve goals, comprising inhibitory control, attention control, working memory, cognitive flexibility, and decision-making [56]. Prior studies have similarly reported that gamified interventions can enhance attention control and cognitive flexibility [20, 24, 57]. This may be attributed to the design elements of GDHIs, such as task-switching, goal-setting, reward mechanisms, and immediate feedback, which enhance children’s cognitive flexibility, attention maintenance, and problem-solving abilities and strengthen executive functions through real-time tasks and dynamic challenges [58, 59].

The meta-analysis indicated that gamified digital health interventions significantly improve motor skills. This improvement may be attributed to the physical interaction and real-time feedback mechanisms integrated into sensor-based games [46, 50], which enable participants to effectively practice coordination and motor skills within a controlled environment. However, contrary to these findings, Jimenez-Muñoz et al. [24] reported no significant effect of video games on gross motor development. Differences in study design (e.g., RCT vs. pretest-posttest without control group) and the limited number of studies available highlight the need for further research with more diverse and larger samples to confirm these effects.

Despite demonstrating positive effects on other outcome measures, the influence of GDHIs on problem behavior improvement was not significant. This finding is consistent with the observations reported by Wang et al. [21], who noted that digital interventions generally have limited effects on behavioral problems. One possible explanation is that the game designs primarily target the enhancement of emotional, social, and cognitive skills rather than directly addressing problem behaviors [33, 36, 37]. Additionally, the complexity and multifactorial nature of behavioral problems may contribute to the limited efficacy. Future research should further investigate the influence of gamified interventions on behavioral problems and explore the potential of integrating multidimensional intervention strategies.

Subgroup analyses revealed that sensor games demonstrated greater efficacy across multiple domains, including emotional skills, social skills, and executive functions, underscoring the significance of the intervention delivery modality. This may be attributed to the fact that sensor games are often designed as highly contextualized and immersive experiences, especially when supported by virtual reality and augmented reality technologies. These games can simulate real-world scenarios by integrating features such as body movement and sensory stimulation, effectively helping children and adolescents with ASD [60]. Nevertheless, the findings of the present study are not in accordance with those of Zhan et al. [23], who determined that the efficacy of game-based digital interventions for mental disorders did not vary by game type. This discrepancy may be related to differences in the characteristics of the target population and the goals of the intervention. Interventions aimed at improving social skills and executive functions in ASD often depend on real-world simulation and multisensory integration, which sensor games are uniquely suited to provide through personalized and interactive experiences. Conversely, Zhan et al.’s study focused on a broader population with mental disorders, where efficacy might rely more on the generality of the intervention content rather than the specific delivery modality. These findings suggest that for interventions for ASD, future research should pay more attention to how to optimize contextual simulation and sensory interactions through advanced technologies to enhance relevance and efficacy. Additionally, the potential influence of game types in various mental health interventions warrants further exploration to better tailor treatments to specific populations and therapeutic goals.

The effectiveness of GDHIs may be underpinned by several neurocognitive mechanisms. First, reinforcement learning theory suggests that reward-based feedback loops embedded in gamified tasks can enhance intrinsic motivation and sustain engagement, thereby supporting learning and behavior change [61]. Second, simulated virtual environments, particularly those delivered via VR and AR, provide opportunities for social cognition training by enabling children to repeatedly practice perspective-taking, emotion recognition, and turn-taking in safe and structured contexts [62]. Third, gamified multitasking elements, such as task-switching and real-time feedback, may modulate executive load by balancing challenge and cognitive resources, which in turn strengthen attention control, working memory, and cognitive flexibility [63]. Finally, sensorimotor feedback integrated into VR/AR-based interventions engages multiple sensory modalities simultaneously, potentially facilitating sensory integration processes that are often impaired in children with ASD [62]. Taken together, these mechanisms provide a theoretical framework explaining why GDHIs may foster improvements across social, emotional, cognitive, and motor domains in children and adolescents with ASD.

In addition to risks related to screen exposure, digital equity represents another major challenge. Children from low-income or rural households may have limited access to devices, reliable internet connectivity, or sufficient power supply and technical support, raising concerns about scalability and fairness [64, 65]. To address these disparities, strategies such as developing low-cost or locally adapted hardware solutions, designing offline-compatible platforms, and implementing school-based or community-center delivery systems could play a pivotal role [66]. Future trials should therefore not only establish clear safety and dosage guidelines but also evaluate strategies to enhance accessibility, thereby balancing therapeutic benefits with potential risks and promoting fair, sustainable implementation.

### Limitations

Notwithstanding the substantial strengths of this study, it is imperative to acknowledge its limitations. Firstly, heterogeneity was observed due to variations in intervention design, outcome measures, and participant characteristics. Although sensitivity and subgroup analyses were performed, these differences may still affect the generalizability and reliability of the findings. Secondly, the overall certainty of evidence was limited. Secondly, the overall certainty of evidence was limited. Most included trials were rated as having high risk of bias or some concerns, and the GRADE ratings predominantly indicated low or very low certainty. Additionally, only a limited number of studies examined improvements in problem behaviors and motor skills, which restricts the generalizability of the results in these areas. Thirdly, although we adopted a broad operational definition of GDHIs to ensure comprehensiveness, the degree of gamification varied across studies. Some interventions were fully game-based, while others incorporated only partial gamified features (e.g., rewards, avatars, or interactive modules embedded in broader digital programs). This variability may limit the precision of our conclusions regarding the specific contribution of gamification itself. Furthermore, most trials had relatively short intervention durations (mean 8.6 weeks), which precluded evaluation of long-term sustainability. Future research should aim to: (1) clearly differentiate between fully gamified and partially gamified interventions; (2) conduct multi-center, large-scale, high-quality randomized controlled trials across diverse cultural and regional contexts; and (3) extend follow-up periods to better determine the durability and translational potential of intervention effects.

### Implications

The findings of this study carry meaningful implications for the application of GDHIs in children and adolescents with ASD. These interventions address key barriers associated with traditional treatments, such as time and location constraints and shortage of professionals, to provide more children and adolescents with ASD with access to treatment, especially in low - and middle-income countries, helping to alleviate the inequalities in access to treatment. This aligns with global efforts to advance digital health, including the World Health Organization’s (WHO) Global Digital Health Strategy and the United Nations’ 3rd Sustainable Development Goal (SDG) emphasizing health equity: “Good Health and Well-Being.”

Beyond these broader public health implications, GDHIs also hold promise for integration into existing therapeutic frameworks. They may complement school-based social skills curricula, provide home-based training modules for parent-mediated interventions, or serve as adjunct tools in clinical rehabilitation programs [62, 67]. Their flexible digital formats allow tailoring to individual needs—for example, visual modules for non-verbal children, or attention-enhancing tasks for those with comorbid ADHD. In low-resource contexts, simplified delivery models such as low-bandwidth mobile applications or school-based group sessions could help overcome infrastructure and cost-related barriers [68].

In light of our findings, future research should concentrate on optimizing intervention designs, tailoring treatments to individual symptom profiles and developmental stages, and evaluating cost-effectiveness. At the same time, it is crucial to consider the potential adverse effects of prolonged screen exposure and to define appropriate intervention “dosages” to balance efficacy and safety. In clinical implementation, healthcare providers should consider multidimensional outcome indicators and individual differences when formulating intervention strategies to maximize therapeutic benefits.

## Conclusions

This systematic review and meta-analysis suggest that gamified digital health interventions show preliminary promise in improving emotional skills, social skills, executive functions, and motor skills in children and adolescents with autism spectrum disorder. However, no significant effects were observed in reducing behavioral problems. Given the predominantly low or very low quality of the included studies, these findings should be interpreted with caution. Future research should focus on developing high-quality, large-scale randomized controlled trials to further evaluate the efficacy, safety, and sustainability of these interventions. While gamified digital health interventions offer an innovative and potentially scalable treatment option, their clinical application requires further validation before widespread implementation.

## Supplementary Information

Below is the link to the electronic supplementary material.

Supplementary Material 1

Supplementary Material 2

Supplementary Material 3

Supplementary Material 4

## Data Availability

The datasets generated and/or analyzed during the current study are available from the corresponding author upon reasonable request.
