# Supplementary material for: Efficacy of gamified digital health interventions for children and adolescents with autism spectrum disorder: a systematic review and meta-analysis
Source: Child Adolesc Psychiatry Ment Health. 2025 Dec 15;20:3. doi: 10.1186/s13034-025-01009-w (PMC12781649; doi:10.1186/s13034-025-01009-w)
Supplement: Supplementary file 1 — Supplementary Material 1 [file 13034_2025_1009_MOESM1_ESM.pdf]

Table Search strategies and database results.

PubMed 2024.11.29	#1	"Autism Spectrum Disorder"[MeSH Terms] OR "Asperger Syndrome"[MeSH Terms] OR "Autistic Disorder"[MeSH Terms]	70757
	#2	((((((((((((((((((((((Autistic Spectrum Disorder[Title/Abstract]) OR (Autistic Spectrum Disorders[Title/Abstract])) OR (Disorder, Autistic Spectrum[Title/Abstract])) OR (Autism Spectrum Disorders[Title/Abstract])) OR (Asperger's Disorder[Title/Abstract])) OR (Aspergers Disorder[Title/Abstract])) OR (Asperger's Syndrome[Title/Abstract])) OR (Aspergers Syndrome[Title/Abstract])) OR (Asperger Disease[Title/Abstract])) OR (Asperger Disorder[Title/Abstract])) OR (Asperger's Disease[Title/Abstract])) OR (Aspergers Disease[Title/Abstract])) OR (Disorder, Autistic[Title/Abstract])) OR (Disorder, Autistic[Title/Abstract])) OR (Disorders, Autistic[Title/Abstract])) OR (Autism[Title/Abstract])) OR (Autism, Early Infantile[Title/Abstract])) OR (Early Infantile Autism[Title/Abstract])) OR (Infantile Autism, Early[Title/Abstract])) OR (Autism, Infantile[Title/Abstract])) OR (Infantile Autism[Title/Abstract])) OR (Kanner's Syndrome[Title/Abstract])) OR (Kanners Syndrome[Title/Abstract])) OR (Kanner Syndrome[Title/Abstract])	76768
	#3	#1 OR #2	76551
	#4	("Video Games"[Mesh]) OR "Exergaming"[Mesh]	8011
	#5	(((((((((((((((Game, Video[Title/Abstract]) OR (Video Game[Title/Abstract])) OR (Computer Game[Title/Abstract])) OR (Game, Computer[Title/Abstract])) OR (Games, Computer[Title/Abstract])) OR (gamifi*[Title/Abstract])) OR (game-based[Title/Abstract])) OR (game[Title/Abstract])) OR (gaming[Title/Abstract])) OR (videogam*[Title/Abstract])) OR (Virtual Reality Exercise[Title/Abstract])) OR (Exercise, Virtual Reality[Title/Abstract])) OR (Virtual Reality Exercises[Title/Abstract])) OR (Active-Video Gaming[Title/Abstract])) OR (Active Video Gaming[Title/Abstract])) OR (Gaming, Active-Video[Title/Abstract])) OR (Exergame[Title/Abstract])	46234
	#6	#4 OR #5	48345
	#7	#3 AND #6	432
Web of science	#1	TS=(Autism Spectrum Disorder OR Asperger Syndrome OR Autistic Disorder OR Autistic Spectrum Disorder OR Autistic Spectrum Disorders OR Disorder, Autistic Spectrum OR Autism Spectrum Disorders OR Asperger's Disorder OR Aspergers Disorder OR Asperger's Syndrome OR Aspergers Syndrome OR Asperger Disease OR Asperger Disorder OR Asperger's Disease OR Aspergers Disease OR Disorder, Autistic OR Disorders, Autistic OR Autism OR Autism,	178519

2024.11.29		Early Infantile OR Early Infantile Autism OR Infantile Autism, Early OR Autism, Infantile OR Infantile Autism OR Kanner's Syndrome OR Kanners Syndrome OR Kanner Syndrome)	
	#2	TS=(Video Games OR Exergaming OR Game, Video OR Video Game OR Computer Game OR Game, Computer OR Games, Computer OR gamifi* OR game-based OR game OR gaming OR videogam* OR exergaming OR Virtual Reality Exercise OR Exercise, Virtual Reality OR Virtual Reality Exercises OR Active-Video Gaming OR Active Video Gaming OR Gaming, Active-Video OR exergames)	469812
	#3	#2AND #1	2215
Cochrane Library 2024.11.29	#1	MeSH descriptor: [Autism Spectrum Disorder] explode all trees	2768
	#2	MeSH descriptor: [Asperger Syndrome] explode all trees	87
	#3	MeSH descriptor: [Autistic Disorder] explode all trees	1654
	#4	(Autistic Spectrum Disorder):ab,ti,kw OR (Autistic Spectrum Disorders):ab,ti,kw OR (Disorder, Autistic Spectrum):ab,ti,kw OR (Autism Spectrum Disorders):ab,ti,kw OR (Asperger's Disorder):ab,ti,kw OR (Aspergers Disorder):ab,ti,kw OR (Asperger's Syndrome):ab,ti,kw OR (Aspergers Syndrome):ab,ti,kw OR (Asperger Disease):ab,ti,kw OR (Asperger Disorder):ab,ti,kw OR (Asperger's Disease):ab,ti,kw OR (Aspergers Disease):ab,ti,kw OR (Disorder, Autistic):ab,ti,kw OR (Disorders, Autistic):ab,ti,kw OR (Autism):ab,ti,kw OR (Autism, Early Infantile):ab,ti,kw OR (Early Infantile Autism):ab,ti,kw OR (Infantile Autism, Early):ab,ti,kw OR (Autism, Infantile):ab,ti,kw OR (Infantile Autism):ab,ti,kw OR (Kanner's Syndrome):ab,ti,kw OR (Kanners Syndrome):ab,ti,kw OR (Kanner Syndrome):ab,ti,kw	5845
	#5	#1OR#2OR#3OR#4	5845
	#6	MeSH descriptor: [Video Games] explode all trees	1272
	#7	MeSH descriptor: [Exergaming] explode all trees	78
	#8	(Video Game):ab,ti,kw OR (Computer Game):ab,ti,kw OR (Game, Computer):ab,ti,kw OR (Games, Computer):ab,ti,kw OR (gamifi*):ab,ti,kw OR (game-based):ab,ti,kw OR (game):ab,ti,kw OR (gaming):ab,ti,kw OR (videogam*):ab,ti,kw OR (Exergamings):ab,ti,kw OR (Virtual Reality Exercise):ab,ti,kw OR	9264

		(Exercise, Virtual Reality):ab,ti,kw OR (Virtual Reality Exercises):ab,ti,kw OR (Active-Video Gaming):ab,ti,kw OR (Active Video Gaming):ab,ti,kw OR (Gaming, Active-Video):ab,ti,kw OR (Exergame):ab,ti,kw	
	#9	#6OR#7OR#8	9433
	#10	#5AND#9	164
EMBASE 2024.11.29	#1	(**'autism'**/exp OR **autism**) AND (**'spectrum'**/exp OR **spectrum**) AND (**'disorder'**/exp OR **disorder**)	59708
	#2	**asperger** AND (**'syndrome'**/exp OR **syndrome**)	5993
	#3	**autistic** AND (**'disorder'**/exp OR **disorder**)	25085
	#4	**'autistic spectrum disorder'**:ab,ti OR **'autistic spectrum disorders'**:ab,ti OR **'disorder, autistic spectrum'**:ab,ti OR **'autism spectrum disorders'**:ab,ti OR **'aspergers disorder'**:ab,ti OR **'aspergers syndrome'**:ab,ti OR **'asperger disease'**:ab,ti OR **'asperger disorder'**:ab,ti OR **'aspergers disease'**:ab,ti OR **'disorder, autistic'**:ab,ti OR **'disorders, autistic'**:ab,ti OR **'autism'**:ab,ti OR **'autism, early infantile'**:ab,ti OR **'early infantile autism'**:ab,ti OR **'infantile autism, early'**:ab,ti OR **'autism, infantile'**:ab,ti OR **'infantile autism'**:ab,ti OR **'kanners syndrome'**:ab,ti OR **'kanner syndrome'**:ab,ti	87385
	#5	(**'video'**/exp OR **video**) AND **games**	7125
	#6	**'exergaming'**/exp OR **exergaming**	1019
	#7	**'video games'**:ab,ti OR **'exergaming'**:ab,ti OR **'game, video'**:ab,ti OR **'video game'**:ab,ti OR **'computer game'**:ab,ti OR **'game, computer'**:ab,ti OR **'games, computer'**:ab,ti OR **'gamifi*'**:ab,ti OR **'game-based'**:ab,ti OR **'game'**:ab,ti OR **'gaming'**:ab,ti OR **'videogam*'**:ab,ti OR **'exergamings'**:ab,ti OR **'virtual reality exercise'**:ab,ti OR **'exercise, virtual reality'**:ab,ti OR **'virtual reality exercises'**:ab,ti OR **'active-video gaming'**:ab,ti OR **'active video gaming'**:ab,ti OR **'gaming, active-video'**:ab,ti OR **'exergame'**:ab,ti	52541
	#8	#1 OR #2 OR #3 OR #4	98061
	#9	#5 OR #6 OR #7	53975

	#10	#8 AND #9	594
Scopus 2024.11.29	#1	TITLE-ABS-KEY ("autism spectrum disorder") OR TITLE-ABS-KEY ("asperger syndrome") OR TITLE-ABS-KEY ("autistic disorder") OR TITLE-ABS-KEY ("autistic spectrum disorder") OR TITLE-ABS-KEY ("autistic spectrum disorders") OR TITLE-ABS-KEY ("disorder, autistic spectrum") OR TITLE-ABS-KEY ("autism spectrum disorders") OR TITLE-ABS-KEY ("asperger's disorder") OR TITLE-ABS-KEY ("aspergers disorder") OR TITLE-ABS-KEY ("asperger's syndrome") OR TITLE-ABS-KEY ("aspergers syndrome") OR TITLE-ABS-KEY ("asperger disease") OR TITLE-ABS-KEY ("asperger disorder") OR TITLE-ABS-KEY ("asperger's disease") OR TITLE-ABS-KEY ("aspergers disease") OR TITLE-ABS-KEY ("disorder, autistic") OR TITLE-ABS-KEY ("disorders, autistic") OR TITLE-ABS-KEY ("autism") OR TITLE-ABS-KEY ("autism, early infantile") OR TITLE-ABS-KEY ("early infantile autism") OR TITLE-ABS-KEY ("infantile autism, early") OR TITLE-ABS-KEY ("autism, infantile") OR TITLE-ABS-KEY ("infantile autism") OR TITLE-ABS-KEY ("kanner's syndrome") OR TITLE-ABS-KEY ("kanners syndrome") OR TITLE-ABS-KEY ("kanner syndrome")	129062
	#2	TITLE-ABS-KEY ("video games") OR TITLE-ABS-KEY ("exergaming ") OR TITLE-ABS-KEY ("game, video") OR TITLE-ABS-KEY ("video game") OR TITLE-ABS-KEY ("computer game") OR TITLE-ABS-KEY ("game, computer") OR TITLE-ABS-KEY ("games, computer") OR TITLE-ABS-KEY ("gamifi*") OR TITLE-ABS-KEY ("game-based") OR TITLE-ABS-KEY ("game") OR TITLE-ABS-KEY ("gaming") OR TITLE-ABS-KEY ("videogam*") OR TITLE-ABS-KEY ("exergamings") OR TITLE-ABS-KEY ("virtual reality exercise") OR TITLE-ABS-KEY ("exercise, virtual reality") OR TITLE-ABS-KEY ("virtual reality exercises") OR TITLE-ABS-KEY ("active-video gaming") OR TITLE-ABS-KEY ("active video gaming") OR TITLE-ABS-KEY ("gaming, active-video") OR TITLE-ABS-KEY ("exergame")	447414
	#3	2AND#1	2110
PsycInfo 2024.11.29	#1	SU (Autism Spectrum Disorder OR Asperger Syndrome OR Autistic Disorder) OR TI (Autism Spectrum Disorder OR Asperger Syndrome OR Autistic Disorder OR Autistic Spectrum Disorder OR Autistic Spectrum Disorders OR Disorder, Autistic Spectrum OR Autism Spectrum Disorders OR Asperger's Disorder OR Aspergers Disorder OR Asperger's Syndrome OR Aspergers Syndrome OR Asperger Disease OR Asperger Disorder OR Asperger's Disease OR Aspergers Disease OR Disorder, Autistic OR Disorders, Autistic OR Autism OR Autism, Early Infantile OR Early Infantile Autism OR Infantile Autism, Early OR Autism, Infantile OR Infantile Autism OR Kanner's Syndrome OR Kanners Syndrome OR Kanner Syndrome) OR AB (Autism Spectrum Disorder OR Asperger Syndrome OR Autistic Disorder OR Autistic Spectrum Disorder OR Autistic Spectrum Disorders OR Disorder, Autistic Spectrum OR Autism Spectrum Disorders OR Asperger's Disorder OR Aspergers Disorder OR Asperger's Syndrome OR Aspergers Syndrome OR Asperger Disease OR Asperger	89,048

		Disorder OR Asperger's Disease OR Aspergers Disease OR Disorder, Autistic OR Disorders, Autistic OR Autism OR Autism, Early Infantile OR Early Infantile Autism OR Infantile Autism, Early OR Autism, Infantile OR Infantile Autism OR Kanner's Syndrome OR Kanners Syndrome OR Kanner Syndrome)	
	#2	SU (Video Games OR Exergaming) OR TI (Video Games OR Exergaming OR Game, Video OR Video Game OR Computer Game OR Game, Computer OR Games, Computer OR gamifi* OR game-based OR game OR gaming OR videogam* OR Exergamings OR Virtual Reality Exercise OR Exercise, Virtual Reality OR Virtual Reality Exercises OR Active-Video Gaming OR Active Video Gaming OR Gaming, Active-Video OR Exergame) OR AB (Video Games OR Exergaming OR Game, Video OR Video Game OR Computer Game OR Game, Computer OR Games, Computer OR gamifi* OR game-based OR game OR gaming OR videogam* OR Exergamings OR Virtual Reality Exercise OR Exercise, Virtual Reality OR Virtual Reality Exercises OR Active-Video Gaming OR Active Video Gaming OR Gaming, Active-Video OR Exergame)	68,832
	#3	#1 AND #2	859
