# Supplementary material for: Efficacy of gamified digital health interventions for children and adolescents with autism spectrum disorder: a systematic review and meta-analysis
Source: Child Adolesc Psychiatry Ment Health. 2025 Dec 15;20:3. doi: 10.1186/s13034-025-01009-w (PMC12781649; doi:10.1186/s13034-025-01009-w)
Supplement: Supplementary file 2 — Supplementary Material 2 [file 13034_2025_1009_MOESM2_ESM.pdf]

Unique ID	1	Study ID	Beaumont et al（2021）
**Domain**	**Signalling question**	**Response**	**Comments**
**Bias arising from the randomization process**	1.1 Was the allocation sequence random?	Y	Source: "These families were then randomly allocated to either the SAS condition (n = 35) or CIA condition (n = 35) using a computerized random number generator."
	1.2 Was the allocation sequence concealed until participants were enrolled and assigned to interventions?	NI	
	1.3 Did baseline differences between intervention groups suggest a problem with the randomization process?	N	Table 1 in the text shows that there were no significant differences between the two groups in terms of baseline characteristics such as age and gender.
	**Risk of bias judgement**	**Some concerns**	
**Bias due to deviations from intended interventions**	2.1.Were participants aware of their assigned intervention during the trial?	PN	Source:"The trial was ‘open-label’ in the sense that participants were not directly informed if they were in the control or treatment group."
	2.2.Were carers and people delivering the interventions aware of participants' assigned intervention during the trial?	PN	The families were trained in intervention, and the parents knew they were assigned to either the SAS group or the CIA group, but they were unaware which group was the intervention group and which was the control group.
	2.3. If Y/PY/NI to 2.1 or 2.2: Were there deviations from the intended intervention that arose because of the experimental context?	NA	
	2.4 If Y/PY to 2.3: Were these deviations likely to have affected the outcome?	NA	
	2.5. If Y/PY/NI to 2.4: Were these deviations from intended intervention balanced between groups?	NA	
	2.6 Was an appropriate analysis used to estimate the effect of assignment to intervention?	Y	The research adopted appropriate statistical methods.
	2.7 If N/PN/NI to 2.6: Was there potential for a substantial impact (on the result) of the failure to analyse participants in the group to which they were randomized?	NA	
	**Risk of bias judgement**	**Low**	
**Bias due to missing outcome data**	3.1 Were data for this outcome available for all, or nearly all, participants randomized?	PN	Source:"Information on the proportion of data missing from outcome measures is summarized in Table 4."
	3.2 If N/PN/NI to 3.1: Is there evidence that result was not biased by missing outcome data?	PN	Although Little’s MCAR test (p = 0.12) suggested that missing values were random, group-level attrition balance was not reported. The use of LOCF for imputation may introduce bias if missingness is related to outcomes.
	3.3 If N/PN to 3.2: Could missingness in the outcome depend on its true value?	N	There is evidence that the absence is random.Source:"Little’s Missing Completely at Random Test suggested that missing values were random"
	3.4 If Y/PY/NI to 3.3: Is it likely that missingness in the outcome depended on its true value?	NA	
	**Risk of bias judgement**	**Low**	
	4.1 Was the method of measuring the outcome inappropriate?	N	The results were obtained using multiple standardized parent rating scales questionnaires.
	4.2 Could measurement or ascertainment of the outcome have differed between intervention groups?	N	Both groups used the same outcome measurement tools.

**Bias in measurement of the outcome**	4.3 Were outcome assessors aware of the intervention received by study participants?	PY	The outcome assessment was conducted in the form of a parent-reported questionnaire. Parents were aware that they had been assigned to either the SAS group or the CIA group, but were not informed which group was the intervention group and which was the control group.
	4.4 If Y/PY/NI to 4.3: Could assessment of the outcome have been influenced by knowledge of intervention received?	NI	
	4.5 If Y/PY/NI to 4.4: Is it likely that assessment of the outcome was influenced by knowledge of intervention received?	NI	
	**Risk of bias judgement**	**High**	
**Bias in selection of the reported result**	5.1 Were the data that produced this result analysed in accordance with a pre-specified analysis plan that was finalized before unblinded outcome data were available for analysis?	Y	The text mentions the "methods/analysis" plan and proceeds in accordance with it.
	5.2 Is the numerical result being assessed likely to have been selected, on the basis of the results, from multiple eligible outcome measurements (e.g. scales, definitions, time points) within the outcome domain?	N	The main and secondary outcomes (parent/teacher ratings) were listed in the methods/abstract. There was no evidence that significant results were selected from multiple measurements for reporting.
	5.3 Is the numerical result being assessed likely to have been selected, on the basis of the results, from multiple eligible analyses of the data?	N	Analysis results of non-selective reporting.
	**Risk of bias judgement**	**Low**	
**Overall bias**	**Risk of bias judgement**	**High**	

**Unique ID**	2	**Study ID**	de Vries et al（2015）
**Domain**	**Signalling question**	**Response**	**Comments**
**Bias arising from the randomization process**	1.1 Was the allocation sequence random?	Y	Source:"One hundred and twenty-one children were randomly (double blind) assigned to the three intervention-conditions."
	1.2 Was the allocation sequence concealed until participants were enrolled and assigned to interventions?	NI	
	1.3 Did baseline differences between intervention groups suggest a problem with the randomization process?	N	Source:"There were no further differences between the three intervention-conditions on screening or pretraining variables."
	**Risk of bias judgement**	**Some concerns**	
	2.1.Were participants aware of their assigned intervention during the trial?	N	Source:"Children and parents were told that there were three intervention-conditions, but not how these conditions were designed."
	2.2.Were carers and people delivering the interventions aware of participants' assigned intervention during the trial?	N	Source:"Parents, children, and assessors were kept blind to intervention-condition until after follow-up."
	2.3. If Y/PY/NI to 2.1 or 2.2: Were there deviations from the intended intervention that arose because of the experimental context?	NA	

**Bias due to deviations from intended interventions**	2.4 If Y/PY to 2.3: Were these deviations likely to have affected the outcome?	NA	
	2.5. If Y/PY/NI to 2.4: Were these deviations from intended intervention balanced between groups?	NA	
	2.6 Was an appropriate analysis used to estimate the effect of assignment to intervention?	Y	The study employed appropriate statistical analysis methods.
	2.7 If N/PN/NI to 2.6: Was there potential for a substantial impact (on the result) of the failure to analyse participants in the group to which they were randomized?	NA	
	**Risk of bias judgement**	**Low**	
**Bias due to missing outcome data**	3.1 Were data for this outcome available for all, or nearly all, participants randomized?	PN	Source:"Attrition-rate was 26%."
	3.2 If N/PN/NI to 3.1: Is there evidence that result was not biased by missing outcome data?	PN	The mechanism of missing reports was not clearly stated.
	3.3 If N/PN to 3.2: Could missingness in the outcome depend on its true value?	NI	
	3.4 If Y/PY/NI to 3.3: Is it likely that missingness in the outcome depended on its true value?	NI	
	**Risk of bias judgement**	**High**	
**Bias in measurement of the outcome**	4.1 Was the method of measuring the outcome inappropriate?	N	A variety of cognitive tests and parent rating scales, all standardized tools, were used.
	4.2 Could measurement or ascertainment of the outcome have differed between intervention groups?	N	Both groups used the same outcome measurement tools.
	4.3 Were outcome assessors aware of the intervention received by study participants?	N	Source:"Parents, children, and assessors were kept blind to intervention-condition until after follow-up."
	4.4 If Y/PY/NI to 4.3: Could assessment of the outcome have been influenced by knowledge of intervention received?	NA	
	4.5 If Y/PY/NI to 4.4: Is it likely that assessment of the outcome was influenced by knowledge of intervention received?	NA	
	**Risk of bias judgement**	**Low**	
**Bias in selection of the reported result**	5.1 Were the data that produced this result analysed in accordance with a pre-specified analysis plan that was finalized before unblinded outcome data were available for analysis?	Y	Analyze as planned.
	5.2 ... multiple eligible outcome measurements (e.g. scales, definitions, time points) within the outcome domain?	N	Did not select significant results from multiple measurement outcomes for reporting.
	5.3 ... multiple eligible analyses of the data?	N	Analysis results of non-selective reporting.
	**Risk of bias judgement**	**Low**	
**Overall bias**	**Risk of bias judgement**	**High**	

Unique ID	3	Study ID	Faja et al（2022）

Domain	Signalling question	Response	Comments
Bias arising from the randomization process	1.1 Was the allocation sequence random?	Y	Source:"Then, children were randomized equally to either active EF training or waitlist control (i.e., parallel design, 1:1 allocation ratio). Randomization order (simple) was computer generated by a staff member not involved with visits and assignments were concealed in sequential, sealed envelopes."
	1.2 Was the allocation sequence concealed until participants were enrolled and assigned to interventions?	Y	
	1.3 Did baseline differences between intervention groups suggest a problem with the randomization process?	N	Table 1 shows that there are no significant differences between the two groups in terms of demographics, symptoms, drug use, IQ, etc.
	**Risk of bias judgement**	**Low**	
Bias due to deviations from intended interventions	2.1.Were participants aware of their assigned intervention during the trial?	PY	The training group knew they were undergoing training, and the waiting group knew they were waiting, so there was a certain degree of awareness. It was not clearly stated whether the participants were blinded.
	2.2.Were carers and people delivering the interventions aware of participants' assigned intervention during the trial?	PY	Intervention providers know the groupings, but evaluators do not:"conducted by staff who were unaware of group status."
	2.3. If Y/PY/NI to 2.1 or 2.2: Were there deviations from the intended intervention that arose because of the experimental context?	NI	
	2.4 If Y/PY to 2.3: Were these deviations likely to have affected the outcome?	NA	
	2.5. If Y/PY/NI to 2.4: Were these deviations from intended intervention balanced between groups?	NA	
	2.6 Was an appropriate analysis used to estimate the effect of assignment to intervention?	Y	The study employed appropriate statistical analysis methods.
	2.7 If N/PN/NI to 2.6: Was there potential for a substantial impact (on the result) of the failure to analyse participants in the group to which they were randomized?	NA	
	**Risk of bias judgement**	**Some concerns**	
Bias due to missing outcome data	3.1 Were data for this outcome available for all, or nearly all, participants randomized?	Y	Reach"all, or nearly all":"All Training participants returned for post-testing. In the Waitlist Group, one family withdrew at randomization and one family was lost to contact. "
	3.2 If N/PN/NI to 3.1: Is there evidence that result was not biased by missing outcome data?	NA	
	3.3 If N/PN to 3.2: Could missingness in the outcome depend on its true value?	NA	
	3.4 If Y/PY/NI to 3.3: Is it likely that missingness in the outcome depended on its true value?	NA	
	**Risk of bias judgement**	**Low**	
	4.1 Was the method of measuring the outcome inappropriate?	N	Standardized tasks (such as the Change task) and questionnaires (such as the RBS-R) were used appropriately.
	4.2 Could measurement or ascertainment of the outcome have differed between intervention groups?	N	Both groups used the same outcome measurement tools.

**Bias in measurement of the outcome**	4.3 Were outcome assessors aware of the intervention received by study participants?	PN	The objective nature of thecognitive tasks vs. the subjective nature of parent reports.
	4.4 If Y/PY/NI to 4.3: Could assessment of the outcome have been influenced by knowledge of intervention received?	NA	
	4.5 If Y/PY/NI to 4.4: Is it likely that assessment of the outcome was influenced by knowledge of intervention received?	NA	
	**Risk of bias judgement**	**Low**	
**Bias in selection of the reported result**	5.1 Were the data that produced this result analysed in accordance with a pre-specified analysis plan that was finalized before unblinded outcome data were available for analysis?	Y	Source:"The study, including selection of outcome measures and analyses, was pre-registered on ClinicalTrials.gov (NCT02361762)."
	5.2 ... multiple eligible outcome measurements (e.g. scales, definitions, time points) within the outcome domain?	N	Did not select significant results from multiple measurement outcomes for reporting.
	5.3 ... multiple eligible analyses of the data?	N	The main analysis and exploratory analysis were conducted, and the effect size was reported.
	**Risk of bias judgement**	**Low**	
**Overall bias**	**Risk of bias judgement**	**Some concerns**	

**Unique ID**	4	**Study ID**	Fletcher-Watson et al (2016)
**Domain**	**Signalling question**	**Response**	**Comments**
**Bias arising from the randomization process**	1.1 Was the allocation sequence random?	Y	Source:"Randomisation was stratified by ADOS social-communication algorithm score... Two randomisation lists were produced using block randomisation with varying and randomly-ordered block sizes."
	1.2 Was the allocation sequence concealed until participants were enrolled and assigned to interventions?	Y	Source:"An independent researcher subsequently produced numbered opaque envelopes containing the random allocation."
	1.3 Did baseline differences between intervention groups suggest a problem with the randomization process?	N	Table 1 shows that there were no significant differences between the two groups in terms of baseline characteristics such as age and gender ($p > 0.05$).
	**Risk of bias judgement**	**Low**	
[illegible]	2.1.Were participants aware of their assigned intervention during the trial?	PY	The intervention group was aware that they were using the iPad application, and the waiting group knew they were waiting. Therefore, there was a certain degree of awareness. It was not clearly stated whether the participants were blinded.
	2.2.Were carers and people delivering the interventions aware of participants' assigned intervention during the trial?	PY	The intervention group knew they were using the iPad application, while the waiting group knew they were waiting.
	2.3. If Y/PY/NI to 2.1 or 2.2: Were there deviations from the intended intervention that arose because of the experimental context?	NI	

**Bias due to deviations from intended interventions**	2.4 If Y/PY to 2.3: Were these deviations likely to have affected the outcome?	NA	
	2.5. If Y/PY/NI to 2.4: Were these deviations from intended intervention balanced between groups?	NA	
	2.6 Was an appropriate analysis used to estimate the effect of assignment to intervention?	Y	The study employed appropriate statistical analysis methods.
	2.7 If N/PN/NI to 2.6: Was there potential for a substantial impact (on the result) of the failure to analyse participants in the group to which they were randomized?	NA	
	**Risk of bias judgement**	**Some concerns**	
**Bias due to missing outcome data**	3.1 Were data for this outcome available for all, or nearly all, participants randomized?	Y	The attrition rate was relatively low (3 in the intervention group and 2 in the control group), reaching "all, or nearly all".
	3.2 If N/PN/NI to 3.1: Is there evidence that result was not biased by missing outcome data?	NA	
	3.3 If N/PN to 3.2: Could missingness in the outcome depend on its true value?	NA	
	3.4 If Y/PY/NI to 3.3: Is it likely that missingness in the outcome depended on its true value?	NA	
	**Risk of bias judgement**	**Low**	
**Bias in measurement of the outcome**	4.1 Was the method of measuring the outcome inappropriate?	N	Standardized tools and the BOSCC observation scale were used, and the methods were appropriate.
	4.2 Could measurement or ascertainment of the outcome have differed between intervention groups?	N	Both groups used the same outcome measurement tools.
	4.3 Were outcome assessors aware of the intervention received by study participants?	N	The primary outcome BOSCC was scored by blinded assessors:"scored by a single, independent researcher... blind to participant group."
	4.4 If Y/PY/NI to 4.3: Could assessment of the outcome have been influenced by knowledge of intervention received?	NA	
	4.5 If Y/PY/NI to 4.4: Is it likely that assessment of the outcome was influenced by knowledge of intervention received?	NA	
	**Risk of bias judgement**	**Low**	
**Bias in selection of the reported result**	5.1 Were the data that produced this result analysed in accordance with a pre-specified analysis plan that was finalized before unblinded outcome data were available for analysis?	Y	Source: "registered with the United Kingdom Clinical Research Network (ID 10428) and on clinicaltrials.gov (ref. NCT01493609)"
	5.2 ... multiple eligible outcome measurements (e.g. scales, definitions, time points) within the outcome domain?	N	Did not select significant results from multiple measurement outcomes for reporting.
	5.3 ... multiple eligible analyses of the data?	N	Inter-group comparisons, correlation analyses and reliable change analyses were conducted.
	**Risk of bias judgement**	**Low**	
**Overall bias**	**Risk of bias judgement**	**Some concerns**	

Unique ID	5	Study ID	Fridenson-Hayo et al（2017）

Domain	Signalling question	Response	Comments
Bias arising from the randomization process	1.1 Was the allocation sequence random?	Y	Source: "Participants were randomly allocated into an intervention group or a treatment as usual control group"
	1.2 Was the allocation sequence concealed until participants were enrolled and assigned to interventions?	NI	
	1.3 Did baseline differences between intervention groups suggest a problem with the randomization process?	N	Table 1 and accompanying text: "the intervention and the waiting-list control groups were comparable locally on age, gender, ADOS-2 scores, and standard scores of two subtests from the Wechsler Scale of Intelligence"
	**Risk of bias judgement**	**Some concerns**	
Bias due to deviations from intended interventions	2.1.Were participants aware of their assigned intervention during the trial?	Y	Source: "The nature of the trial (waitlist control) makes blinding impossible for participants."
	2.2.Were carers and people delivering the interventions aware of participants' assigned intervention during the trial?	PY	Parents were required to supervise their children's use of the software (intervention group) or were informed that they were in a waiting period (control group). The researchers were responsible for introducing the software and conducting follow-up visits for monitoring, so they should be aware of the group allocation.
	2.3. If Y/PY/NI to 2.1 or 2.2: Were there deviations from the intended intervention that arose because of the experimental context?	PY	Source: "In Israel, when participants missed whole weeks of software use (e.g., due to a family vacation, or illness), they were given additional time. In addition, when the monitoring calls reveled participants need more time to complete the intervention, they were given up to four extra weeks. In Sweden, additional time was given only if participants missed whole weeks of software use, but not in order to complete the intervention."
	2.4 If Y/PY to 2.3: Were these deviations likely to have affected the outcome?	PY	One of the primary outcome measures was the parents' report (SRS-2, VABS). The parents' expectations and level of involvement are likely to influence their assessment of their children's behavior (i.e., placebo-by-proxy effect), and the authors explicitly mentioned this possibility in the discussion section. "It is also possible... that the lack of replication... stems from a placebo-by-proxy effect [68], which altered the parents' perception of their children's behavior"
	2.5. If Y/PY/NI to 2.4: Were these deviations from intended intervention balanced between groups?	NI	
	2.6 Was an appropriate analysis used to estimate the effect of assignment to intervention?	Y	The study employed appropriate statistical analysis methods.
	2.7 If N/PN/NI to 2.6: Was there potential for a substantial impact (on the result) of the failure to analyse participants in the group to which they were randomized?	NA	
	**Risk of bias judgement**	**High**	
Bias due to	3.1 Were data for this outcome available for all, or nearly all, participants randomized?	PN	Figure 5 (Flow diagram) and text: "nine participants from the intervention groups had failed to complete the entire training period"
	3.2 If N/PN/NI to 3.1: Is there evidence that result was not biased by missing outcome data?	Y	Source: "Participants who dropped out of the intervention group and those who had completed the program did not differ on age, IQ, ADOS, VABS, parental education and the pre-intervention ER task scores."

**missing outcome data**	3.3 If N/PN to 3.2: Could missingness in the outcome depend on its true value?	NA	
	3.4 If Y/PY/NI to 3.3: Is it likely that missingness in the outcome depended on its true value?	NA	
	**Risk of bias judgement**	**Low**	
**Bias in measurement of the outcome**	4.1 Was the method of measuring the outcome inappropriate?	N	A variety of cognitive tests and parent rating scales, all standardized tools, were used.
	4.2 Could measurement or ascertainment of the outcome have differed between intervention groups?	N	Both groups used the same outcome measurement tools.
	4.3 Were outcome assessors aware of the intervention received by study participants?	PN	The objective nature of the ER tasks vs. the subjective nature of parent reports.
	4.4 If Y/PY/NI to 4.3: Could assessment of the outcome have been influenced by knowledge of intervention received?	NA	
	4.5 If Y/PY/NI to 4.4: Is it likely that assessment of the outcome was influenced by knowledge of intervention received?	NA	
	**Risk of bias judgement**	**Low**	
**Bias in selection of the reported result**	5.1 Were the data that produced this result analysed in accordance with a pre-specified analysis plan that was finalized before unblinded outcome data were available for analysis?	Y	Analyze as planned.
	5.2 ... multiple eligible outcome measurements (e.g. scales, definitions, time points) within the outcome domain?	N	Did not select significant results from multiple measurement outcomes for reporting.
	5.3 ... multiple eligible analyses of the data?	N	Analysis results of non-selective reporting.
	**Risk of bias judgement**	**Low**	
**Overall bias**	**Risk of bias judgement**	**High**	

**Unique ID**	6	**Study ID**	Griffin et al（2021）
**Domain**	**Signalling question**	**Response**	**Comments**
**Bias arising from the randomization process**	1.1 Was the allocation sequence random?	Y	Source:"Randomization proceeded according to a computer-generated list in a 1:1 ratio..., stratified by gender and full-scale IQ."
	1.2 Was the allocation sequence concealed until participants were enrolled and assigned to interventions?	NI	
	1.3 Did baseline differences between intervention groups suggest a problem with the randomization process?	N	Table 1: "Sample characteristics"

	**Risk of bias judgement**	**Some concerns**	
**Bias due to deviations from intended interventions**	2.1.Were participants aware of their assigned intervention during the trial?	Y	Source:"Parents and adolescent participants were not blinded from knowing the condition assignment."
	2.2.Were carers and people delivering the interventions aware of participants' assigned intervention during the trial?	Y	Parents were responsible for supervising game use, and researchers were responsible for fidelity intervention, both being aware of the group assignment.
	2.3. If Y/PY/NI to 2.1 or 2.2: Were there deviations from the intended intervention that arose because of the experimental context?	NI	
	2.4 If Y/PY to 2.3: Were these deviations likely to have affected the outcome?	NA	
	2.5. If Y/PY/NI to 2.4: Were these deviations from intended intervention balanced between groups?	NA	
	2.6 Was an appropriate analysis used to estimate the effect of assignment to intervention?	Y	The study employed appropriate statistical analysis methods.
	2.7 If N/PN/NI to 2.6: Was there potential for a substantial impact (on the result) of the failure to analyse participants in the group to which they were randomized?	NA	
	**Risk of bias judgement**	**Some concerns**	
**Bias due to missing outcome data**	3.1 Were data for this outcome available for all, or nearly all, participants randomized?	Y	All randomized participants completed the pre- and post-test evaluations, with no missing data.
	3.2 If N/PN/NI to 3.1: Is there evidence that result was not biased by missing outcome data?	NA	
	3.3 If N/PN to 3.2: Could missingness in the outcome depend on its true value?	NA	
	3.4 If Y/PY/NI to 3.3: Is it likely that missingness in the outcome depended on its true value?	NA	
	**Risk of bias judgement**	**Low**	
**Bias in measurement of the outcome**	4.1 Was the method of measuring the outcome inappropriate?	N	The Gaze Perception task, which was specially designed, and the standardized parent report scales (SSIS, SRS-2) were used appropriately.
	4.2 Could measurement or ascertainment of the outcome have differed between intervention groups?	N	Both groups used the same outcome measurement tools.
	4.3 Were outcome assessors aware of the intervention received by study participants?	PN	For the Gaze Perception task: N, standardized computer task, consistent for all participants.<br>For the parent report: PN, parents were not blinded, but no reporting bias was found in the text.
	4.4 If Y/PY/NI to 4.3: Could assessment of the outcome have been influenced by knowledge of intervention received?	NA	
	4.5 If Y/PY/NI to 4.4: Is it likely that assessment of the outcome was influenced by knowledge of intervention received?	NA	
	**Risk of bias judgement**	**Low**	

**Bias in selection of the reported result**	5.1 Were the data that produced this result analysed in accordance with a pre-specified analysis plan that was finalized before unblinded outcome data were available for analysis?	Y	Source:"The protocol for this RCT has been published... and is also registered through clinicaltrials.gov"
	5.2 ... multiple eligible outcome measurements (e.g. scales, definitions, time points) within the outcome domain?	N	Did not select significant results from multiple measurement outcomes for reporting.
	5.3 ... multiple eligible analyses of the data?	N	Analysis results of non-selective reporting.
	**Risk of bias judgement**	**Low**	
**Overall bias**	**Risk of bias judgement**	**Some concerns**	

**Unique ID**	7	**Study ID**	Hopkins et al（2011）
**Domain**	**Signalling question**	**Response**	**Comments**
**Bias arising from the randomization process**	1.1 Was the allocation sequence random?	Y	Source:"The children were randomly assigned to the training group or the control group."
	1.2 Was the allocation sequence concealed until participants were enrolled and assigned to interventions?	NI	
	1.3 Did baseline differences between intervention groups suggest a problem with the randomization process?	N	Source:"No significant differences were found between the groups."
	**Risk of bias judgement**	**Some concerns**	
**Bias due to deviations from intended interventions**	2.1.Were participants aware of their assigned intervention during the trial?	PN	Since both the intervention group and the control group used game software, participants might not have been aware whether they were in the intervention group or the control group.
	2.2.Were carers and people delivering the interventions aware of participants' assigned intervention during the trial?	N	Source:"The parents were blind to their child’s group assignment"
	2.3. If Y/PY/NI to 2.1 or 2.2: Were there deviations from the intended intervention that arose because of the experimental context?	NA	
	2.4 If Y/PY to 2.3: Were these deviations likely to have affected the outcome?	NA	
	2.5. If Y/PY/NI to 2.4: Were these deviations from intended intervention balanced between groups?	NA	
	2.6 Was an appropriate analysis used to estimate the effect of assignment to intervention?	Y	The study employed appropriate statistical analysis methods.
	2.7 If N/PN/NI to 2.6: Was there potential for a substantial impact (on the result) of the failure to analyse participants in the group to which they were randomized?	NA	
	**Risk of bias judgement**	**Low**	

**Bias due to missing outcome data**	3.1 Were data for this outcome available for all, or nearly all, participants randomized?	Y	Initially, 51 children were enrolled, but 2 were excluded due to low attendance, and 49 completed the entire study:"Forty-nine children with LFA or HFA completed the project."
	3.2 If N/PN/NI to 3.1: Is there evidence that result was not biased by missing outcome data?	NA	
	3.3 If N/PN to 3.2: Could missingness in the outcome depend on its true value?	NA	
	3.4 If Y/PY/NI to 3.3: Is it likely that missingness in the outcome depended on its true value?	NA	
	**Risk of bias judgement**	**Low**	
**Bias in measurement of the outcome**	4.1 Was the method of measuring the outcome inappropriate?	N	Standardized tools and structured observations were used appropriately.
	4.2 Could measurement or ascertainment of the outcome have differed between intervention groups?	N	Both groups used the same outcome measurement tools.
	4.3 Were outcome assessors aware of the intervention received by study participants?	N	For objective tasks (such as the Emotion Recognition Test): Y, consistent for all participants.<br>For parent reports (such as the SSRS): Y, parents blinded to group assignment.<br>For observations (such as the SSO): Y, observers blinded to group assignment.
	4.4 If Y/PY/NI to 4.3: Could assessment of the outcome have been influenced by knowledge of intervention received?	NA	
	4.5 If Y/PY/NI to 4.4: Is it likely that assessment of the outcome was influenced by knowledge of intervention received?	NA	
	**Risk of bias judgement**	**Low**	
**Bias in selection of the reported result**	5.1 Were the data that produced this result analysed in accordance with a pre-specified analysis plan that was finalized before unblinded outcome data were available for analysis?	PY	Analyze as planned.
	5.2 ... multiple eligible outcome measurements (e.g. scales, definitions, time points) within the outcome domain?	N	Did not select significant results from multiple measurement outcomes for reporting.
	5.3 ... multiple eligible analyses of the data?	N	Analysis results of non-selective reporting.
	**Risk of bias judgement**	**Low**	
**Overall bias**	**Risk of bias judgement**	**Some concerns**	

Unique ID	8	Study ID	Kirst et al（2022）
**Domain**	**Signalling question**	**Response**	**Comments**
**Bias arising from the randomization process**	1.1 Was the allocation sequence random?	Y	Source:"Eligible participants were randomized to either the TG or the active control group (CG). Minimization was performed with a randomization ratio of 0.8 and the two three-staged stratification factors verbal age and study center using MinimPy ."
	1.2 Was the allocation sequence concealed until participants were enrolled and assigned to interventions?	NI	
	1.3 Did baseline differences between intervention groups suggest a problem with the randomization process?	PY	Source:"At baseline, the groups differed significantly in autism symptoms, as measured by SCQ, with the TG showing higher symptomatology (TG: M = 22.8, SD = 6.2; CG: M = 19.5, SD = 6.2; t (78) = 2.39, p = .019, d = 0.54)."
	**Risk of bias judgement**	**High**	
**Bias due to deviations from intended interventions**	2.1.Were participants aware of their assigned intervention during the trial?	Y	Source:"Since ZE is an online psychotherapy with a clear focus on socio-emotional skills, blinding children and caretakers was not possible."
	2.2.Were carers and people delivering the interventions aware of participants' assigned intervention during the trial?	Y	
	2.3. If Y/PY/NI to 2.1 or 2.2: Were there deviations from the intended intervention that arose because of the experimental context?	N	Source:"Treatment fidelity was highly rated in both groups (TG: M = 4.32, SD = 0.77; CG: M = 4.07, SD = 0.87; t(59) = 1.19, p = .240)."
	2.4 If Y/PY to 2.3: Were these deviations likely to have affected the outcome?	NA	
	2.5. If Y/PY/NI to 2.4: Were these deviations from intended intervention balanced between groups?	NA	
	2.6 Was an appropriate analysis used to estimate the effect of assignment to intervention?	Y	The study employed appropriate statistical analysis methods.
	2.7 If N/PN/NI to 2.6: Was there potential for a substantial impact (on the result) of the failure to analyse participants in the group to which they were randomized?	NA	
	**Risk of bias judgement**	**Low**	
**Bias due to missing outcome data**	3.1 Were data for this outcome available for all, or nearly all, participants randomized?	PN	Source:"In total, 40 GEM datasets were missing across all time points (16.7%)."
	3.2 If N/PN/NI to 3.1: Is there evidence that result was not biased by missing outcome data?	PN	The article employed FIML to handle missing data, but there is no direct evidence to prove that the missingness is related to the true values of the outcomes.
	3.3 If N/PN to 3.2: Could missingness in the outcome depend on its true value?	NI	
	3.4 If Y/PY/NI to 3.3: Is it likely that missingness in the outcome depended on its true value?	NI	
	**Risk of bias judgement**	**High**	
	4.1 Was the method of measuring the outcome inappropriate?	N	Standardized scales were used and the methods were appropriate.
	4.2 Could measurement or ascertainment of the outcome have differed between intervention groups?	N	Both groups used the same outcome measurement tools.

**Bias in measurement of the outcome**	4.3 Were outcome assessors aware of the intervention received by study participants?	PY	The failure to blind the study might have led to the assessors knowing the group assignments.
	4.4 If Y/PY/NI to 4.3: Could assessment of the outcome have been influenced by knowledge of intervention received?	PY	Since the evaluators may be aware of the groupings, there may be assessment bias.
	4.5 If Y/PY/NI to 4.4: Is it likely that assessment of the outcome was influenced by knowledge of intervention received?	N	Source:"the behavioral results from the objective tests support the parents' and teachers' subjective observations, it is unlikely that the effects are solely attributes to observer bias."
	**Risk of bias judgement**	**Some concerns**	
**Bias in selection of the reported result**	5.1 Were the data that produced this result analysed in accordance with a pre-specified analysis plan that was finalized before unblinded outcome data were available for analysis?	Y	Source:"The registered study protocol was fulfilled except for one outcome measure being excluded from analysis..."
	5.2 ... multiple eligible outcome measurements (e.g. scales, definitions, time points) within the outcome domain?	N	Did not select significant results from multiple measurement outcomes for reporting.
	5.3 ... multiple eligible analyses of the data?	N	Analysis results of non-selective reporting.
	**Risk of bias judgement**	**Low**	
**Overall bias**	**Risk of bias judgement**	**High**	

**Unique ID**	9	**Study ID**	Macoun et al（2021）
**Domain**	**Signalling question**	**Response**	**Comments**
**Bias arising from the randomization process**	1.1 Was the allocation sequence random?	Y	Source:"This study utilized a waitlist-control design with random assignment to either an intervention (CQ) or waitlist-control group."
	1.2 Was the allocation sequence concealed until participants were enrolled and assigned to interventions?	NI	
	1.3 Did baseline differences between intervention groups suggest a problem with the randomization process?	N	Source:"The intervention and control groups did not differ significantly with respect to gender, age, diagnostic comorbidities, ASD symptom levels, or attention/EF problems (see Table 1)."
	**Risk of bias judgement**	**Some concerns**	
	2.1.Were participants aware of their assigned intervention during the trial?	PY	The training group knew they were undergoing training, and the waiting group knew they were waiting, so there was a certain degree of awareness. It was not clearly stated whether the participants were blinded.
	2.2.Were carers and people delivering the interventions aware of participants' assigned intervention during the trial?	PY	Because it was a waiting list control group design.
[illegible]	2.3. If Y/PY/NI to 2.1 or 2.2: Were there deviations from the intended intervention that arose because of the experimental context?	N	Source:"Fidelity was demonstrated by the RA via completion of quizzes... weekly check-ins... and observations by the senior research team."

Bias due to deviations from intended interventions	2.4 If Y/PY to 2.3: Were these deviations likely to have affected the outcome?	NA	
	2.5. If Y/PY/NI to 2.4: Were these deviations from intended intervention balanced between groups?	NA	
	2.6 Was an appropriate analysis used to estimate the effect of assignment to intervention?	Y	The study employed appropriate statistical analysis methods.
	2.7 If N/PN/NI to 2.6: Was there potential for a substantial impact (on the result) of the failure to analyse participants in the group to which they were randomized?	NA	
	**Risk of bias judgement**	**Low**	
Bias due to missing outcome data	3.1 Were data for this outcome available for all, or nearly all, participants randomized?	PN	Source:"Due to technical challenges, the following data loss occurred: 7 from WJ-III Math Fluency (2 pre/5 post), 5 from ORF reading fluency (1 pre/4 post), 7 from the Colored Boxes task (2 pre/5 post) and 1 from the KiTAP (1 post)."
	3.2 If N/PN/NI to 3.1: Is there evidence that result was not biased by missing outcome data?	PN	The author did not conduct any investigation into the missing mechanism, we cannot rule out this possibility.
	3.3 If N/PN to 3.2: Could missingness in the outcome depend on its true value?	NI	
	3.4 If Y/PY/NI to 3.3: Is it likely that missingness in the outcome depended on its true value?	NI	
	**Risk of bias judgement**	**High**	
Bias in measurement of the outcome	4.1 Was the method of measuring the outcome inappropriate?	N	Standardized tests were used and the methods were appropriate.
	4.2 Could measurement or ascertainment of the outcome have differed between intervention groups?	N	Both groups used the same outcome measurement tools.
	4.3 Were outcome assessors aware of the intervention received by study participants?	N	Objective task measurement using computers
	4.4 If Y/PY/NI to 4.3: Could assessment of the outcome have been influenced by knowledge of intervention received?	NA	
	4.5 If Y/PY/NI to 4.4: Is it likely that assessment of the outcome was influenced by knowledge of intervention received?	NA	
	**Risk of bias judgement**	**Low**	
Bias in selection of the reported result	5.1 Were the data that produced this result analysed in accordance with a pre-specified analysis plan that was finalized before unblinded outcome data were available for analysis?	Y	Analyze as planned.
	5.2 ... multiple eligible outcome measurements (e.g. scales, definitions, time points) within the outcome domain?	PN	Did not select significant results from multiple measurement outcomes for reporting.
	5.3 ... multiple eligible analyses of the data?	N	Analysis results of non-selective reporting.
	**Risk of bias judgement**	**Low**	
Overall bias	**Risk of bias judgement**	**High**	

Unique ID	10	Study ID	Mercado et al（2021）

Domain	Signalling question	Response	Comments
**Bias arising from the randomization process**	1.1 Was the allocation sequence random?	Y	Source:"We randomly assigned the 26 participants into two groups of equal size (i.e., the experimental group and the control group)."
	1.2 Was the allocation sequence concealed until participants were enrolled and assigned to interventions?	NI	
	1.3 Did baseline differences between intervention groups suggest a problem with the randomization process?	N	There were no significant differences reported between the two groups at baseline, and the sample characteristics were similar.
	**Risk of bias judgement**	**Some concerns**	
**Bias due to deviations from intended interventions**	2.1.Were participants aware of their assigned intervention during the trial?	PY	The intervention group played FarmerKeeper, while the control group watched cartoons. Participants might have known their group assignment.
	2.2.Were carers and people delivering the interventions aware of participants' assigned intervention during the trial?	PY	The intervention group played FarmerKeeper, while the control group watched cartoons. The therapist might have known the group allocation.
	2.3. If Y/PY/NI to 2.1 or 2.2: Were there deviations from the intended intervention that arose because of the experimental context?	N	Source:"All neurofeedback sessions were video-recorded... to complement the self-reports"
	2.4 If Y/PY to 2.3: Were these deviations likely to have affected the outcome?	NA	
	2.5. If Y/PY/NI to 2.4: Were these deviations from intended intervention balanced between groups?	NA	
	2.6 Was an appropriate analysis used to estimate the effect of assignment to intervention?	Y	The study employed appropriate statistical analysis methods.
	2.7 If N/PN/NI to 2.6: Was there potential for a substantial impact (on the result) of the failure to analyse participants in the group to which they were randomized?	NA	
	**Risk of bias judgement**	**Low**	
**Bias due to missing outcome data**	3.1 Were data for this outcome available for all, or nearly all, participants randomized?	PY	The attrition rate is relatively low:"we had to remove data from three participants, two from the control group and one from the experimental group"
	3.2 If N/PN/NI to 3.1: Is there evidence that result was not biased by missing outcome data?	NA	
	3.3 If N/PN to 3.2: Could missingness in the outcome depend on its true value?	NA	
	3.4 If Y/PY/NI to 3.3: Is it likely that missingness in the outcome depended on its true value?	NA	
	**Risk of bias judgement**	**Low**	
	4.1 Was the method of measuring the outcome inappropriate?	N	The use of standardized scales and EEG data is reasonable in terms of methodology.
	4.2 Could measurement or ascertainment of the outcome have differed between intervention groups?	N	Both groups used the same outcome measurement tools.

**Bias in measurement of the outcome**	4.3 Were outcome assessors aware of the intervention received by study participants?	PN	The EEG data is objective and not affected by the grouping, but there may be some doubts about the scales.
	4.4 If Y/PY/NI to 4.3: Could assessment of the outcome have been influenced by knowledge of intervention received?	NA	
	4.5 If Y/PY/NI to 4.4: Is it likely that assessment of the outcome was influenced by knowledge of intervention received?	NA	
	**Risk of bias judgement**	**Low**	
**Bias in selection of the reported result**	5.1 Were the data that produced this result analysed in accordance with a pre-specified analysis plan that was finalized before unblinded outcome data were available for analysis?	Y	Analyze as planned.
	5.2 ... multiple eligible outcome measurements (e.g. scales, definitions, time points) within the outcome domain?	N	Did not select significant results from multiple measurement outcomes for reporting.
	5.3 ... multiple eligible analyses of the data?	N	Analysis results of non-selective reporting.
	**Risk of bias judgement**	**Low**	
**Overall bias**	**Risk of bias judgement**	**Some concerns**	

**Unique ID**	11	**Study ID**	Nekar et al（2022)

**Domain**	**Signalling question**	**Response**	**Comments**
**Bias arising from the randomization process**	1.1 Was the allocation sequence random?	Y	Source:"Participants were randomly allocated to two groups... The allocation was conducted by an independent investigator prior to the beginning of the study by producing 24 random numbers."
	1.2 Was the allocation sequence concealed until participants were enrolled and assigned to interventions?	Y	Source:"The allocation sequence was concealed using a sealed opaque envelope."
	1.3 Did baseline differences between intervention groups suggest a problem with the randomization process?	N	Source:"There were no statistically significant differences in age, height, weight, or mental status between the intervention and control groups at baseline..."
	**Risk of bias judgement**	**Low**	
**Bias due to deviations from**	2.1.Were participants aware of their assigned intervention during the trial?	N	Source:"Participants did not receive any explanation about how the different groups would perform the training in order to be blind to the type of intervention. "
	2.2.Were carers and people delivering the interventions aware of participants' assigned intervention during the trial?	N	
	2.3. If Y/PY/NI to 2.1 or 2.2: Were there deviations from the intended intervention that arose because of the experimental context?	NA	
	2.4 If Y/PY to 2.3: Were these deviations likely to have affected the outcome?	NA	

**intended interventions**	2.5. If Y/PY/NI to 2.4: Were these deviations from intended intervention balanced between groups?	NA	
	2.6 Was an appropriate analysis used to estimate the effect of assignment to intervention?	Y	The study employed appropriate statistical analysis methods.
	2.7 If N/PN/NI to 2.6: Was there potential for a substantial impact (on the result) of the failure to analyse participants in the group to which they were randomized?	NA	
	**Risk of bias judgement**	**Low**	
**Bias due to missing outcome data**	3.1 Were data for this outcome available for all, or nearly all, participants randomized?	Y	Source:"All participants... were included in the analysis."
	3.2 If N/PN/NI to 3.1: Is there evidence that result was not biased by missing outcome data?	NA	
	3.3 If N/PN to 3.2: Could missingness in the outcome depend on its true value?	NA	
	3.4 If Y/PY/NI to 3.3: Is it likely that missingness in the outcome depended on its true value?	NA	
	**Risk of bias judgement**	**Low**	
**Bias in measurement of the outcome**	4.1 Was the method of measuring the outcome inappropriate?	N	Standardized tools were used and the methods were appropriate.
	4.2 Could measurement or ascertainment of the outcome have differed between intervention groups?	N	Both groups used the same outcome measurement tools.
	4.3 Were outcome assessors aware of the intervention received by study participants?	PN	Computerized task measurement is objective and not affected by grouping, but there may be some doubts about the scale.
	4.4 If Y/PY/NI to 4.3: Could assessment of the outcome have been influenced by knowledge of intervention received?	NA	
	4.5 If Y/PY/NI to 4.4: Is it likely that assessment of the outcome was influenced by knowledge of intervention received?	NA	
	**Risk of bias judgement**	**Low**	
**Bias in selection of the reported result**	5.1 Were the data that produced this result analysed in accordance with a pre-specified analysis plan that was finalized before unblinded outcome data were available for analysis?	Y	Analyze as planned.
	5.2 ... multiple eligible outcome measurements (e.g. scales, definitions, time points) within the outcome domain?	N	Did not select significant results from multiple measurement outcomes for reporting.
	5.3 ... multiple eligible analyses of the data?	N	Analysis results of non-selective reporting.
	**Risk of bias judgement**	**Low**	
**Overall bias**	**Risk of bias judgement**	**Low**	

Unique ID	12	Study ID	Rice et al（2015)

Domain	Signalling question	Response	Comments
**Bias arising from the randomization process**	1.1 Was the allocation sequence random?	Y	Source:"After all participants were recruited, they were randomly assigned to a study group."
	1.2 Was the allocation sequence concealed until participants were enrolled and assigned to interventions?	NI	
	1.3 Did baseline differences between intervention groups suggest a problem with the randomization process?	N	Source:"There were no statistically significant differences in age, height, weight, or mental status between the intervention and control groups at baseline..."
	**Risk of bias judgement**	**Some concerns**	
**Bias due to deviations from intended interventions**	2.1.Were participants aware of their assigned intervention during the trial?	PN	Both the experimental group (FaceSay™ program) and the control group (SuccessMaker® program) utilized game software, so the participants might not have been aware whether they were in the intervention group or the control group.
	2.2.Were carers and people delivering the interventions aware of participants' assigned intervention during the trial?	N	Source:"Teachers were blinded to the participants' training group membership..."
	2.3. If Y/PY/NI to 2.1 or 2.2: Were there deviations from the intended intervention that arose because of the experimental context?	NA	
	2.4 If Y/PY to 2.3: Were these deviations likely to have affected the outcome?	NA	
	2.5. If Y/PY/NI to 2.4: Were these deviations from intended intervention balanced between groups?	NA	
	2.6 Was an appropriate analysis used to estimate the effect of assignment to intervention?	Y	The study employed appropriate statistical analysis methods.
	2.7 If N/PN/NI to 2.6: Was there potential for a substantial impact (on the result) of the failure to analyse participants in the group to which they were randomized?	NA	
	**Risk of bias judgement**	**Low**	
**Bias due to missing outcome data**	3.1 Were data for this outcome available for all, or nearly all, participants randomized?	Y	Source:"There were no dropouts during the experiment."
	3.2 If N/PN/NI to 3.1: Is there evidence that result was not biased by missing outcome data?	NA	
	3.3 If N/PN to 3.2: Could missingness in the outcome depend on its true value?	NA	
	3.4 If Y/PY/NI to 3.3: Is it likely that missingness in the outcome depended on its true value?	NA	
	**Risk of bias judgement**	**Low**	
	4.1 Was the method of measuring the outcome inappropriate?	N	Standardized tools were used and the methods were appropriate.
	4.2 Could measurement or ascertainment of the outcome have differed between intervention groups?	N	Both groups used the same outcome measurement tools.

**Bias in measurement of the outcome**	4.3 Were outcome assessors aware of the intervention received by study participants?	N	Source:"Teachers were blinded to the participants' training group membership..."
	4.4 If Y/PY/NI to 4.3: Could assessment of the outcome have been influenced by knowledge of intervention received?	NA	
	4.5 If Y/PY/NI to 4.4: Is it likely that assessment of the outcome was influenced by knowledge of intervention received?	NA	
	**Risk of bias judgement**	**Low**	
**Bias in selection of the reported result**	5.1 Were the data that produced this result analysed in accordance with a pre-specified analysis plan that was finalized before unblinded outcome data were available for analysis?	Y	Analyze as planned.
	5.2 ... multiple eligible outcome measurements (e.g. scales, definitions, time points) within the outcome domain?	N	Did not select significant results from multiple measurement outcomes for reporting.
	5.3 ... multiple eligible analyses of the data?	N	Analysis results of non-selective reporting.
	**Risk of bias judgement**	**Low**	
**Overall bias**	**Risk of bias judgement**	**Some concerns**	

**Unique ID**	13	**Study ID**	Sepehri Bonab et al (2024)

**Domain**	**Signalling question**	**Response**	**Comments**
**Bias arising from the randomization process**	1.1 Was the allocation sequence random?	Y	Source:"The selected participants were randomly assigned into the experimental (n = 20) and control (n = 20) groups by the researcher."
	1.2 Was the allocation sequence concealed until participants were enrolled and assigned to interventions?	NI	
	1.3 Did baseline differences between intervention groups suggest a problem with the randomization process?	N	Source:"There were no statistically significant differences in the demographic characteristics of participants between the two groups at baseline."
	**Risk of bias judgement**	**Some concerns**	
**Bias due to deviations from**	2.1.Were participants aware of their assigned intervention during the trial?	Y	The research design led to informed group assignment.
	2.2.Were carers and people delivering the interventions aware of participants' assigned intervention during the trial?	Y	The research design led to informed group assignment.
	2.3. If Y/PY/NI to 2.1 or 2.2: Were there deviations from the intended intervention that arose because of the experimental context?	PN	
	2.4 If Y/PY to 2.3: Were these deviations likely to have affected the outcome?	NA	

**intended interventions**	2.5. If Y/PY/NI to 2.4: Were these deviations from intended intervention balanced between groups?	NA	
	2.6 Was an appropriate analysis used to estimate the effect of assignment to intervention?	Y	The study employed appropriate statistical analysis methods.
	2.7 If N/PN/NI to 2.6: Was there potential for a substantial impact (on the result) of the failure to analyse participants in the group to which they were randomized?	NA	
	**Risk of bias judgement**	**Low**	
**Bias due to missing outcome data**	3.1 Were data for this outcome available for all, or nearly all, participants randomized?	Y	Source:"All participants completed the intervention without dropouts"
	3.2 If N/PN/NI to 3.1: Is there evidence that result was not biased by missing outcome data?	NA	
	3.3 If N/PN to 3.2: Could missingness in the outcome depend on its true value?	NA	
	3.4 If Y/PY/NI to 3.3: Is it likely that missingness in the outcome depended on its true value?	NA	
	**Risk of bias judgement**	**Low**	
**Bias in measurement of the outcome**	4.1 Was the method of measuring the outcome inappropriate?	N	Standardized tools were used and the methods were appropriate.
	4.2 Could measurement or ascertainment of the outcome have differed between intervention groups?	N	Both groups used the same outcome measurement tools.
	4.3 Were outcome assessors aware of the intervention received by study participants?	PN	Computerized task measurement is objective and not affected by grouping, but there may be some doubts about the scale.
	4.4 If Y/PY/NI to 4.3: Could assessment of the outcome have been influenced by knowledge of intervention received?	NA	
	4.5 If Y/PY/NI to 4.4: Is it likely that assessment of the outcome was influenced by knowledge of intervention received?	NA	
	**Risk of bias judgement**	**Low**	
**Bias in selection of the reported result**	5.1 Were the data that produced this result analysed in accordance with a pre-specified analysis plan that was finalized before unblinded outcome data were available for analysis?	Y	Analyze as planned.
	5.2 ... multiple eligible outcome measurements (e.g. scales, definitions, time points) within the outcome domain?	N	Did not select significant results from multiple measurement outcomes for reporting.
	5.3 ... multiple eligible analyses of the data?	N	Analysis results of non-selective reporting.
	**Risk of bias judgement**	**Low**	
**Overall bias**	**Risk of bias judgement**	**Some concerns**	

Unique ID	14	Study ID	Soles-Núñez et al（2024）

Domain	Signalling question	Response	Comments
Bias arising from the randomization process	1.1 Was the allocation sequence random?	Y	Source:"randomly allocated 30 to the control group (CG) and 30 to the experimental group (GE)"
	1.2 Was the allocation sequence concealed until participants were enrolled and assigned to interventions?	NI	
	1.3 Did baseline differences between intervention groups suggest a problem with the randomization process?	N	No baseline differences were reported in the text, but the random allocation process was clear, so it is assumed that there were no significant differences by default:"two groups were formed... randomly assigned"
	**Risk of bias judgement**	**Some concerns**	
Bias due to deviations from intended interventions	2.1.Were participants aware of their assigned intervention during the trial?	PY	The research design may lead to possible informed consent issues.
	2.2.Were carers and people delivering the interventions aware of participants' assigned intervention during the trial?	PY	The research design may lead to possible informed consent issues.
	2.3. If Y/PY/NI to 2.1 or 2.2: Were there deviations from the intended intervention that arose because of the experimental context?	PN	
	2.4 If Y/PY to 2.3: Were these deviations likely to have affected the outcome?	NA	
	2.5. If Y/PY/NI to 2.4: Were these deviations from intended intervention balanced between groups?	NA	
	2.6 Was an appropriate analysis used to estimate the effect of assignment to intervention?	Y	The study employed appropriate statistical analysis methods.
	2.7 If N/PN/NI to 2.6: Was there potential for a substantial impact (on the result) of the failure to analyse participants in the group to which they were randomized?	NA	
	**Risk of bias judgement**	**Low**	
Bias due to missing outcome data	3.1 Were data for this outcome available for all, or nearly all, participants randomized?	Y	There was no dropout data, and all participants completed both the pre- and post-tests.
	3.2 If N/PN/NI to 3.1: Is there evidence that result was not biased by missing outcome data?	NA	
	3.3 If N/PN to 3.2: Could missingness in the outcome depend on its true value?	NA	
	3.4 If Y/PY/NI to 3.3: Is it likely that missingness in the outcome depended on its true value?	NA	
	**Risk of bias judgement**	**Low**	
	4.1 Was the method of measuring the outcome inappropriate?	N	Standardized tools were used and the methods were appropriate.
	4.2 Could measurement or ascertainment of the outcome have differed between intervention groups?	N	Both groups used the same outcome measurement tools.

**Bias in measurement of the outcome**	4.3 Were outcome assessors aware of the intervention received by study participants?	NI	
	4.4 If Y/PY/NI to 4.3: Could assessment of the outcome have been influenced by knowledge of intervention received?	NI	
	4.5 If Y/PY/NI to 4.4: Is it likely that assessment of the outcome was influenced by knowledge of intervention received?	NI	
	**Risk of bias judgement**	**High**	
**Bias in selection of the reported result**	5.1 Were the data that produced this result analysed in accordance with a pre-specified analysis plan that was finalized before unblinded outcome data were available for analysis?	Y	Analyze as planned.
	5.2 ... multiple eligible outcome measurements (e.g. scales, definitions, time points) within the outcome domain?	N	Did not select significant results from multiple measurement outcomes for reporting.
	5.3 ... multiple eligible analyses of the data?	N	Analysis results of non-selective reporting.
	**Risk of bias judgement**	**Low**	
**Overall bias**	**Risk of bias judgement**	**High**	

**Unique ID**	15	**Study ID**	Soniyasri et al （2024）

**Domain**	**Signalling question**	**Response**	**Comments**
**Bias arising from the randomization process**	1.1 Was the allocation sequence random?	Y	Source:"The participants were selected from special education centre Kamal Deep using a concealed envelope method for random allocation were utilized in this research study."
	1.2 Was the allocation sequence concealed until participants were enrolled and assigned to interventions?	Y	
	1.3 Did baseline differences between intervention groups suggest a problem with the randomization process?	N	Table 2 shows that there was no significant difference between the two groups in the pretest scores of TGMD-2 and PBS.
	**Risk of bias judgement**	**Low**	
**Bias due to deviations from**	2.1.Were participants aware of their assigned intervention during the trial?	PY	The research design may lead to possible informed consent issues.
	2.2.Were carers and people delivering the interventions aware of participants' assigned intervention during the trial?	PY	
	2.3. If Y/PY/NI to 2.1 or 2.2: Were there deviations from the intended intervention that arose because of the experimental context?	NI	
	2.4 If Y/PY to 2.3: Were these deviations likely to have affected the outcome?	NA	

**intended interventions**	2.5. If Y/PY/NI to 2.4: Were these deviations from intended intervention balanced between groups?	NA	
	2.6 Was an appropriate analysis used to estimate the effect of assignment to intervention?	Y	The study employed appropriate statistical analysis methods.
	2.7 If N/PN/NI to 2.6: Was there potential for a substantial impact (on the result) of the failure to analyse participants in the group to which they were randomized?	NA	
	**Risk of bias judgement**	**Some concerns**	
**Bias due to missing outcome data**	3.1 Were data for this outcome available for all, or nearly all, participants randomized?	Y	Each group consisted of 10 people, all of whom completed the intervention and assessment with no missing data.
	3.2 If N/PN/NI to 3.1: Is there evidence that result was not biased by missing outcome data?	NA	
	3.3 If N/PN to 3.2: Could missingness in the outcome depend on its true value?	NA	
	3.4 If Y/PY/NI to 3.3: Is it likely that missingness in the outcome depended on its true value?	NA	
	**Risk of bias judgement**	**Low**	
**Bias in measurement of the outcome**	4.1 Was the method of measuring the outcome inappropriate?	N	Standardized tools were used and the methods were appropriate.
	4.2 Could measurement or ascertainment of the outcome have differed between intervention groups?	N	Both groups used the same outcome measurement tools.
	4.3 Were outcome assessors aware of the intervention received by study participants?	PY	The research design may lead to possible informed consent issues.
	4.4 If Y/PY/NI to 4.3: Could assessment of the outcome have been influenced by knowledge of intervention received?	NI	
	4.5 If Y/PY/NI to 4.4: Is it likely that assessment of the outcome was influenced by knowledge of intervention received?	NI	
	**Risk of bias judgement**	**High**	
**Bias in selection of the reported result**	5.1 Were the data that produced this result analysed in accordance with a pre-specified analysis plan that was finalized before unblinded outcome data were available for analysis?	Y	Analyze as planned.
	5.2 ... multiple eligible outcome measurements (e.g. scales, definitions, time points) within the outcome domain?	N	Did not select significant results from multiple measurement outcomes for reporting.
	5.3 ... multiple eligible analyses of the data?	N	Analysis results of non-selective reporting.
	**Risk of bias judgement**	**Low**	
**Overall bias**	**Risk of bias judgement**	**High**	

Unique ID	16	Study ID	Sosnowski et al（2022）
**Domain**	**Signalling question**	**Response**	**Comments**
**Bias arising from the randomization process**	1.1 Was the allocation sequence random?	Y	Source:"children were randomly assigned to either the intervention (n = 25) or control (n = 29) condition"
	1.2 Was the allocation sequence concealed until participants were enrolled and assigned to interventions?	NI	
	1.3 Did baseline differences between intervention groups suggest a problem with the randomization process?	N	Source:"No significant differences were found in age, gender, Ekman-60, or PPVT-4 scores between groups at baseline, and no outliers were detected for baseline Ekman-60 scores; all data were approximately normally distributed"
	**Risk of bias judgement**	**Some concerns**	
**Bias due to deviations from intended interventions**	2.1.Were participants aware of their assigned intervention during the trial?	N	Source:"Participants, monitors, assessment administrators, and staff completing measures were blinded to group assignment."
	2.2.Were carers and people delivering the interventions aware of participants' assigned intervention during the trial?	N	
	2.3. If Y/PY/NI to 2.1 or 2.2: Were there deviations from the intended intervention that arose because of the experimental context?	NA	
	2.4 If Y/PY to 2.3: Were these deviations likely to have affected the outcome?	NA	
	2.5. If Y/PY/NI to 2.4: Were these deviations from intended intervention balanced between groups?	NA	
	2.6 Was an appropriate analysis used to estimate the effect of assignment to intervention?	Y	The study employed appropriate statistical analysis methods.
	2.7 If N/PN/NI to 2.6: Was there potential for a substantial impact (on the result) of the failure to analyse participants in the group to which they were randomized?	NA	
	**Risk of bias judgement**	**Low**	
**Bias due to missing outcome data**	3.1 Were data for this outcome available for all, or nearly all, participants randomized?	PN	Source:"We used last observation carried forward (LOCF) to impute missing values for the four participants who were lost to follow-up after the baseline Ekman-60 assessment. This imputation method was used given the small amount of missing data and lack of auxiliary data (e.g., IQ, symptom severity) to generate accurate values that account for the missing data pattern in more sophisticated, stochastic imputation methods." There is a possibility of bias due to missing outcome data.
	3.2 If N/PN/NI to 3.1: Is there evidence that result was not biased by missing outcome data?	PN	
	3.3 If N/PN to 3.2: Could missingness in the outcome depend on its true value?	NI	
	3.4 If Y/PY/NI to 3.3: Is it likely that missingness in the outcome depended on its true value?	NI	
	**Risk of bias judgement**	**High**	
	4.1 Was the method of measuring the outcome inappropriate?	N	Standardized tools were used and the methods were appropriate.

**Bias in measurement of the outcome**	4.2 Could measurement or ascertainment of the outcome have differed between intervention groups?	N	Both groups used the same outcome measurement tools.
	4.3 Were outcome assessors aware of the intervention received by study participants?	N	Source:"Participants, monitors, assessment administrators, and staff completing measures were blinded to group assignment."
	4.4 If Y/PY/NI to 4.3: Could assessment of the outcome have been influenced by knowledge of intervention received?	NA	
	4.5 If Y/PY/NI to 4.4: Is it likely that assessment of the outcome was influenced by knowledge of intervention received?	NA	
	**Risk of bias judgement**	**Low**	
**Bias in selection of the reported result**	5.1 Were the data that produced this result analysed in accordance with a pre-specified analysis plan that was finalized before unblinded outcome data were available for analysis?	Y	Analyze as planned.
	5.2 ... multiple eligible outcome measurements (e.g. scales, definitions, time points) within the outcome domain?	N	Did not select significant results from multiple measurement outcomes for reporting.
	5.3 ... multiple eligible analyses of the data?	N	Analysis results of non-selective reporting.
	**Risk of bias judgement**	**Low**	
**Overall bias**	**Risk of bias judgement**	**High**	

**Unique ID**	17	**Study ID**	van den Berk-Smeekens et al（2022）

**Domain**	**Signalling question**	**Response**	**Comments**
**Bias arising from the randomization process**	1.1 Was the allocation sequence random?	Y	Source: "Participants were randomly assigned to either PRT, robot-assisted PRT or TAU (1:1:1)"
	1.2 Was the allocation sequence concealed until participants were enrolled and assigned to interventions?	NI	
	1.3 Did baseline differences between intervention groups suggest a problem with the randomization process?	N	Table 1 shows no significant differences in baseline characteristics (all $p > 0.05$).
	**Risk of bias judgement**	**Some concerns**	
	2.1.Were participants aware of their assigned intervention during the trial?	PY	The research design may lead to possible informed consent issues.
	2.2.Were carers and people delivering the interventions aware of participants' assigned intervention during the trial?	PY	
	2.3. If Y/PY/NI to 2.1 or 2.2: Were there deviations from the intended intervention that arose because of the experimental context?	NI	

**Bias due to deviations from intended interventions**	2.4 If Y/PY to 2.3: Were these deviations likely to have affected the outcome?	NA	
	2.5. If Y/PY/NI to 2.4: Were these deviations from intended intervention balanced between groups?	NA	
	2.6 Was an appropriate analysis used to estimate the effect of assignment to intervention?	Y	The study employed appropriate statistical analysis methods.
	2.7 If N/PN/NI to 2.6: Was there potential for a substantial impact (on the result) of the failure to analyse participants in the group to which they were randomized?	NA	
	**Risk of bias judgement**	**Some concerns**	
**Bias due to missing outcome data**	3.1 Were data for this outcome available for all, or nearly all, participants randomized?	PY	There is data missing, but the proportion of missing data is relatively low, and the distribution is similar among groups.
	3.2 If N/PN/NI to 3.1: Is there evidence that result was not biased by missing outcome data?	NA	
	3.3 If N/PN to 3.2: Could missingness in the outcome depend on its true value?	NA	
	3.4 If Y/PY/NI to 3.3: Is it likely that missingness in the outcome depended on its true value?	NA	
	**Risk of bias judgement**	**Low**	
**Bias in measurement of the outcome**	4.1 Was the method of measuring the outcome inappropriate?	N	Standardized tools were used and the methods were appropriate.
	4.2 Could measurement or ascertainment of the outcome have differed between intervention groups?	N	Both groups used the same outcome measurement tools.
	4.3 Were outcome assessors aware of the intervention received by study participants?	N	Evaluated by blinded assessors("blinded to treatment allocation").
	4.4 If Y/PY/NI to 4.3: Could assessment of the outcome have been influenced by knowledge of intervention received?	NA	
	4.5 If Y/PY/NI to 4.4: Is it likely that assessment of the outcome was influenced by knowledge of intervention received?	NA	
	**Risk of bias judgement**	**Low**	
**Bias in selection of the reported result**	5.1 Were the data that produced this result analysed in accordance with a pre-specified analysis plan that was finalized before unblinded outcome data were available for analysis?	Y	Analyze as planned.
	5.2 ... multiple eligible outcome measurements (e.g. scales, definitions, time points) within the outcome domain?	N	Did not select significant results from multiple measurement outcomes for reporting.
	5.3 ... multiple eligible analyses of the data?	N	Analysis results of non-selective reporting.
	**Risk of bias judgement**	**Low**	
**Overall bias**	**Risk of bias judgement**	**Some concerns**	

Unique ID	18	Study ID	Vasilevska Petrovska et al （2019）
**Domain**	**Signalling question**	**Response**	**Comments**
**Bias arising from the randomization process**	1.1 Was the allocation sequence random?	Y	Source: "Participants were randomly assigned to either an intervention or a control group."
	1.2 Was the allocation sequence concealed until participants were enrolled and assigned to interventions?	NI	
	1.3 Did baseline differences between intervention groups suggest a problem with the randomization process?	N	Source: "As shown in Table 2 significant between group differences were not found on age, CARS scores or ECT sores."
	**Risk of bias judgement**	**Some concerns**	
**Bias due to deviations from intended interventions**	2.1.Were participants aware of their assigned intervention during the trial?	PY	The research design may lead to possible informed consent issues.
	2.2.Were carers and people delivering the interventions aware of participants' assigned intervention during the trial?	PY	
	2.3. If Y/PY/NI to 2.1 or 2.2: Were there deviations from the intended intervention that arose because of the experimental context?	NI	
	2.4 If Y/PY to 2.3: Were these deviations likely to have affected the outcome?	NA	
	2.5. If Y/PY/NI to 2.4: Were these deviations from intended intervention balanced between groups?	NA	
	2.6 Was an appropriate analysis used to estimate the effect of assignment to intervention?	Y	The study employed appropriate statistical analysis methods.
	2.7 If N/PN/NI to 2.6: Was there potential for a substantial impact (on the result) of the failure to analyse participants in the group to which they were randomized?	NA	
	**Risk of bias judgement**	**Some concerns**	
**Bias due to missing outcome data**	3.1 Were data for this outcome available for all, or nearly all, participants randomized?	Y	Initially, 33 people were randomly selected. One person did not complete the intervention. Eventually, all 32 people were included in the analysis.
	3.2 If N/PN/NI to 3.1: Is there evidence that result was not biased by missing outcome data?	NA	
	3.3 If N/PN to 3.2: Could missingness in the outcome depend on its true value?	NA	
	3.4 If Y/PY/NI to 3.3: Is it likely that missingness in the outcome depended on its true value?	NA	
	**Risk of bias judgement**	**Low**	
	4.1 Was the method of measuring the outcome inappropriate?	N	Standardized tools were used and the methods were appropriate.
	4.2 Could measurement or ascertainment of the outcome have differed between intervention groups?	N	Both groups used the same outcome measurement tools.

**Bias in measurement of the outcome**	4.3 Were outcome assessors aware of the intervention received by study participants?	N	Result evaluation is a computer task.
	4.4 If Y/PY/NI to 4.3: Could assessment of the outcome have been influenced by knowledge of intervention received?	NA	
	4.5 If Y/PY/NI to 4.4: Is it likely that assessment of the outcome was influenced by knowledge of intervention received?	NA	
	**Risk of bias judgement**	**Low**	
**Bias in selection of the reported result**	5.1 Were the data that produced this result analysed in accordance with a pre-specified analysis plan that was finalized before unblinded outcome data were available for analysis?	Y	Analyze as planned.
	5.2 ... multiple eligible outcome measurements (e.g. scales, definitions, time points) within the outcome domain?	N	Did not select significant results from multiple measurement outcomes for reporting.
	5.3 ... multiple eligible analyses of the data?	N	Analysis results of non-selective reporting.
	**Risk of bias judgement**	**Low**	
**Overall bias**	**Risk of bias judgement**	**Some concerns**	

**Unique ID**	19	**Study ID**	Vukićević et al（2019）

**Domain**	**Signalling question**	**Response**	**Comments**
**Bias arising from the randomization process**	1.1 Was the allocation sequence random?	Y	Source: "We randomly assigned participants equally (five participants to a group) to experimental and control groups."
	1.2 Was the allocation sequence concealed until participants were enrolled and assigned to interventions?	NI	
	1.3 Did baseline differences between intervention groups suggest a problem with the randomization process?	N	Source:"Statistical tests of any significant differences between the experimental and control group found the groups were not different with regard to age."
	**Risk of bias judgement**	**Some concerns**	
**Bias due to deviations from**	2.1.Were participants aware of their assigned intervention during the trial?	Y	The research design led to informed grouping.
	2.2.Were carers and people delivering the interventions aware of participants' assigned intervention during the trial?	Y	
	2.3. If Y/PY/NI to 2.1 or 2.2: Were there deviations from the intended intervention that arose because of the experimental context?	NI	
	2.4 If Y/PY to 2.3: Were these deviations likely to have affected the outcome?	NA	

**intended interventions**	2.5. If Y/PY/NI to 2.4: Were these deviations from intended intervention balanced between groups?	NA	
	2.6 Was an appropriate analysis used to estimate the effect of assignment to intervention?	Y	The study employed appropriate statistical analysis methods.
	2.7 If N/PN/NI to 2.6: Was there potential for a substantial impact (on the result) of the failure to analyse participants in the group to which they were randomized?	NA	
	**Risk of bias judgement**	**Some concerns**	
**Bias due to missing outcome data**	3.1 Were data for this outcome available for all, or nearly all, participants randomized?	Y	All participants completed the study.
	3.2 If N/PN/NI to 3.1: Is there evidence that result was not biased by missing outcome data?	NA	
	3.3 If N/PN to 3.2: Could missingness in the outcome depend on its true value?	NA	
	3.4 If Y/PY/NI to 3.3: Is it likely that missingness in the outcome depended on its true value?	NA	
	**Risk of bias judgement**	**Low**	
**Bias in measurement of the outcome**	4.1 Was the method of measuring the outcome inappropriate?	N	Use standardized tools and methods appropriately.
	4.2 Could measurement or ascertainment of the outcome have differed between intervention groups?	N	Both groups used the same outcome measurement tools.
	4.3 Were outcome assessors aware of the intervention received by study participants?	PY	Educators may be aware of the grouping situation and participate in the assessment.
	4.4 If Y/PY/NI to 4.3: Could assessment of the outcome have been influenced by knowledge of intervention received?	NI	
	4.5 If Y/PY/NI to 4.4: Is it likely that assessment of the outcome was influenced by knowledge of intervention received?	NI	
	**Risk of bias judgement**	**High**	
**Bias in selection of the reported result**	5.1 Were the data that produced this result analysed in accordance with a pre-specified analysis plan that was finalized before unblinded outcome data were available for analysis?	Y	Analyze as planned.
	5.2 ... multiple eligible outcome measurements (e.g. scales, definitions, time points) within the outcome domain?	N	Did not select significant results from multiple measurement outcomes for reporting.
	5.3 ... multiple eligible analyses of the data?	N	Analysis results of non-selective reporting.
	**Risk of bias judgement**	**Low**	
**Overall bias**	**Risk of bias judgement**	**High**	

Unique ID	20	Study ID	Wijnhoven et al（2020）

Domain	Signalling question	Response	Comments
Bias arising from the randomization process	1.1 Was the allocation sequence random?	Y	Source:"Randomization was performed by an independent researcher and was carried out separately for each location of the different institutes (n = 6), using a computerized random number generator. Randomization was stratified by gender and age (8–11 and 12–15 years old)."
	1.2 Was the allocation sequence concealed until participants were enrolled and assigned to interventions?	NI	
	1.3 Did baseline differences between intervention groups suggest a problem with the randomization process?	N	Source:"No significant differences were found between both conditions in age, TIQ, gender, education of the child and parental marital status."
	**Risk of bias judgement**	**Some concerns**	
Bias due to deviations from intended interventions	2.1.Were participants aware of their assigned intervention during the trial?	N	Source:"To minimize the chance of finding placebo-effects of Mindlight, children in the control condition received the computer game 'Triple Town'."
	2.2.Were carers and people delivering the interventions aware of participants' assigned intervention during the trial?	N	
	2.3. If Y/PY/NI to 2.1 or 2.2: Were there deviations from the intended intervention that arose because of the experimental context?	NA	
	2.4 If Y/PY to 2.3: Were these deviations likely to have affected the outcome?	NA	
	2.5. If Y/PY/NI to 2.4: Were these deviations from intended intervention balanced between groups?	NA	
	2.6 Was an appropriate analysis used to estimate the effect of assignment to intervention?	Y	The study employed appropriate statistical analysis methods.
	2.7 If N/PN/NI to 2.6: Was there potential for a substantial impact (on the result) of the failure to analyse participants in the group to which they were randomized?	NA	
	**Risk of bias judgement**	**Low**	
Bias due to missing outcome data	3.1 Were data for this outcome available for all, or nearly all, participants randomized?	PN	There are some missing data. The expected sample size was 120, but the final sample size was 109.
	3.2 If N/PN/NI to 3.1: Is there evidence that result was not biased by missing outcome data?	PN	The study used FIML for analysis but provided no direct evidence that the missing data are random or balanced across groups. Therefore, there is a possibility of bias arising from the missing data.
	3.3 If N/PN to 3.2: Could missingness in the outcome depend on its true value?	NI	
	3.4 If Y/PY/NI to 3.3: Is it likely that missingness in the outcome depended on its true value?	NI	
	**Risk of bias judgement**	**High**	
	4.1 Was the method of measuring the outcome inappropriate?	N	Use standardized tools and methods appropriately.
	4.2 Could measurement or ascertainment of the outcome have differed between intervention groups?	N	Both groups used the same outcome measurement tools.

**Bias in measurement of the outcome**	4.3 Were outcome assessors aware of the intervention received by study participants?	N	The primary outcome was a self-assessment questionnaire completed by the participants, who were unaware of their group assignment.
	4.4 If Y/PY/NI to 4.3: Could assessment of the outcome have been influenced by knowledge of intervention received?	NA	
	4.5 If Y/PY/NI to 4.4: Is it likely that assessment of the outcome was influenced by knowledge of intervention received?	NA	
	**Risk of bias judgement**	**Low**	
**Bias in selection of the reported result**	5.1 Were the data that produced this result analysed in accordance with a pre-specified analysis plan that was finalized before unblinded outcome data were available for analysis?	Y	Analyze as planned.
	5.2 ... multiple eligible outcome measurements (e.g. scales, definitions, time points) within the outcome domain?	N	Did not select significant results from multiple measurement outcomes for reporting.
	5.3 ... multiple eligible analyses of the data?	N	Analysis results of non-selective reporting.
	**Risk of bias judgement**	**Low**	
**Overall bias**	**Risk of bias judgement**	**High**	

**Unique ID**	21	**Study ID**	Zhao et al（2022）
**Domain**	**Signalling question**	**Response**	**Comments**
**Bias arising from the randomization process**	1.1 Was the allocation sequence random?	PN	Source:"Patients participating in the intervention were selected according to their admission order. The researchers numbered the patients according to the time of admission. Patients with odd numbers were included in the intervention group, and patients with even numbers were included in the control group."
	1.2 Was the allocation sequence concealed until participants were enrolled and assigned to interventions?	PN	
	1.3 Did baseline differences between intervention groups suggest a problem with the randomization process?	N	Source:"There were no significant differences in age, gender, or treatment duration between the two groups (all P > 0.05)."
	**Risk of bias judgement**	**High**	
**Bias due to deviations from [illegible]**	2.1.Were participants aware of their assigned intervention during the trial?	PY	The research design led to a possible situation of informed group assignment.
	2.2.Were carers and people delivering the interventions aware of participants' assigned intervention during the trial?	PY	
	2.3. If Y/PY/NI to 2.1 or 2.2: Were there deviations from the intended intervention that arose because of the experimental context?	PY	Source:"During the intervention, several children refused to wear head mounted VR displays... ‘ desensitization therapy’ was adopted..."
	2.4 If Y/PY to 2.3: Were these deviations likely to have affected the outcome?	N	Although adjustments were made, the researchers maintained the intervention through desensitization therapy, which did not significantly affect the final outcome.

intended interventions	2.5. If Y/PY/NI to 2.4: Were these deviations from intended intervention balanced between groups?	NA	
	2.6 Was an appropriate analysis used to estimate the effect of assignment to intervention?	Y	The study employed appropriate statistical analysis methods.
	2.7 If N/PN/NI to 2.6: Was there potential for a substantial impact (on the result) of the failure to analyse participants in the group to which they were randomized?	NA	
	**Risk of bias judgement**	**Some concerns**	
**Bias due to missing outcome data**	3.1 Were data for this outcome available for all, or nearly all, participants randomized?	Y	Initially, there were 47 people, and finally, 44 people (93.6%) remained.
	3.2 If N/PN/NI to 3.1: Is there evidence that result was not biased by missing outcome data?	NA	
	3.3 If N/PN to 3.2: Could missingness in the outcome depend on its true value?	NA	
	3.4 If Y/PY/NI to 3.3: Is it likely that missingness in the outcome depended on its true value?	NA	
	**Risk of bias judgement**	**Low**	
**Bias in measurement of the outcome**	4.1 Was the method of measuring the outcome inappropriate?	N	Use standardized tools and methods appropriately.
	4.2 Could measurement or ascertainment of the outcome have differed between intervention groups?	N	Both groups used the same outcome measurement tools.
	4.3 Were outcome assessors aware of the intervention received by study participants?	PY	The research design led to a possible situation of informed group assignment.
	4.4 If Y/PY/NI to 4.3: Could assessment of the outcome have been influenced by knowledge of intervention received?	NI	
	4.5 If Y/PY/NI to 4.4: Is it likely that assessment of the outcome was influenced by knowledge of intervention received?	NI	
	**Risk of bias judgement**	**High**	
**Bias in selection of the reported result**	5.1 Were the data that produced this result analysed in accordance with a pre-specified analysis plan that was finalized before unblinded outcome data were available for analysis?	Y	Analyze as planned.
	5.2 ... multiple eligible outcome measurements (e.g. scales, definitions, time points) within the outcome domain?	N	Did not select significant results from multiple measurement outcomes for reporting.
	5.3 ... multiple eligible analyses of the data?	N	Analysis results of non-selective reporting.
	**Risk of bias judgement**	**Low**	
**Overall bias**	**Risk of bias judgement**	**High**	
