# Supplementary material for: Efficacy of gamified digital health interventions for children and adolescents with autism spectrum disorder: a systematic review and meta-analysis
Source: Child Adolesc Psychiatry Ment Health. 2025 Dec 15;20:3. doi: 10.1186/s13034-025-01009-w (PMC12781649; doi:10.1186/s13034-025-01009-w)
Supplement: Supplementary file 3 — Supplementary Material 3 [file 13034_2025_1009_MOESM3_ESM.pdf]

Comparison of Original and Revised Overall Risk of Bias Judgments with Affected Domains and Justifications

This table summarizes the revisions made to the overall RoB 2.0 judgments following reviewer feedback. The most affected domains and concise rationales for each change are presented for transparency and traceability.

Study ID	Original Judgment	Revised Judgment	Domains most affected	Rationale
Beaumont et al（2021）	Low	High	D4	Parent-reported outcomes were not blinded, increasing risk of expectancy bias.
de Vries et al（2015）	Low	High	D3	Substantial attrition without sufficient evidence that missing data were random or balanced.
Faja et al（2022）	Some concerns	Some concerns		
Fletcher-Watson et al（2016）	Some concerns	Some concerns		
Fridenson-Hayo et al（2017）	High	High		
Griffin et al（2021）	Low	Some concerns	D1	Allocation concealment procedures were not documented, warranting downgrade.
Hopkins et al（2011	Low	Some concerns	D1	Allocation concealment procedures were not documented, warranting downgrade.
Kirst et al（2022）	Some concerns	High	D1,D3	Allocation concealment procedures unclear and missing data handling not adequately justified.
Macoun et al（2021）	Some concerns	High	D3	Substantial attrition without sufficient evidence that missing data were random or balanced.
Mercado et al（2021）	Some concerns	Some concerns		

Nekar et al（2022）	Low	Low		
Rice et al（2015）	Low	Some concerns	D1	Allocation concealment procedures were not documented, warranting downgrade.
Sepehri Bonab et al（2024）	Some concerns	Some concerns		
Soles-Núñez et al（2024）	Some concerns	High	D4	Lack of assessor blinding for self-/parent-rated outcomes likely influenced results.
Soniyasri et al（2024）	Some concerns	High	D4	Measurement bias likely as assessors were aware of group assignment.
Sosnowski et al（2022）	Low	High	D3	Substantial attrition without sufficient evidence that missing data were random or balanced.
van den Berk-Smeekens et al（2022）	Some concerns	Some concerns		
Vasilevska Petrovska et al（2019）	Some concerns	Some concerns		
Vukićević et al（2019）	Some concerns	High	D4	Measurement bias likely as assessors were aware of group assignment.
Wijnhoven et al（2020）	Low	High	D3	Missing outcome data not fully justified; FIML analysis insufficient for bias exclusion.
Zhao et al（2022）	High	High		
