# Supplementary material for: Efficacy of gamified digital health interventions for children and adolescents with autism spectrum disorder: a systematic review and meta-analysis
Source: Child Adolesc Psychiatry Ment Health. 2025 Dec 15;20:3. doi: 10.1186/s13034-025-01009-w (PMC12781649; doi:10.1186/s13034-025-01009-w)
Supplement: Supplementary file 4 — Supplementary Material 4 [file 13034_2025_1009_MOESM4_ESM.pdf]

Table 1. Outcome measures, informants, and classification of subjective versus objective assessments included in the meta-analysis

	Study	Outcome measures	Informant	Subjective/Objective
**Emotional skills**	Beaumont et al(2021)	Spence Children’s Anxiety Scale (SCAS)	Parent	Subjective (proxy-report)
	Fridenson-Hayo et al(2017)	Emotion recognition tasks-Face task	Child	Objective (performance-based)
	Griffin et al(2021)	Gaze Perception task	Child	Objective (performance-based)
	Hopkins et al(2011)	Emotion Recognition Test	Child	Objective (performance-based)
	Rice et al(2015)	Emotion/Affect Recognition (AR)-NEPSY-II Affect Recognition subtest	Child	Objective (standardized cognitive test)
	Sepehri Bonab et al(2024)	Emotion Regulation Checklist(ERC)- ER	Parent	Subjective (proxy-report)
	Sosnowski et al(2022)	Electronic Ekman-60	Child	Objective (performance-based)
	Vasilevska Petrovska et al(2019)	Emotion Comprehension Test (ECT)-Face task	Child	Objective (performance-based)
	Wijnhoven et al(2020)	Spence Children’s Anxiety Scale (SCAS)	Child (self-report)	Subjective (self-report)
	Zhao et al(2022)	Psychoeducational Profile, Third Edition (PEP-3)-Emotional expression	Clinician	Clinician-rated (semi-structured)
**Social skills**	Beaumont et al(2021)	Social Skills Questionnaire (SSQ)	Parent	Subjective (proxy-report)
	Faja et al(2022)	Social Skills Improvement System(SSIS)	Parent	Subjective (proxy-report)
	Griffin et al(2021)	Social Responsiveness Scale, second edition (SRS-2)	Parent	Subjective (proxy-report)
	Hopkins et al(2011)	Social Skills Rating System (SSRS)	Parent	Subjective (proxy-report)
	Rice et al(2015)	Social Responsiveness Scale, second edition (SRS-2)	Teacher	Subjective (proxy-report)
	van den Berk-Smeekens et al(2022)	Social Responsiveness Scale(SRS-2)	Parent	Subjective (proxy-report)
	Zhao et al(2022)	Psychoeducational Profile, Third Edition (PEP-3)-Social interaction	Clinician	Clinician-rated (semi-structured)
**Executive functions**	de Vries et al(2015)	Inhibition:Stop signal reaction time (SSRT)	Child	Objective (performance-based)
	Faja et al(2022)	Change Task-stop signal reaction time (SSRT)	Child	Objective (performance-based)
	Macoun et al(2021)	Test of Attentional Performance Children’s Version (KiTAP)-Selective Attention (Ghosts Total errors)	Child	Objective (performance-based)
	Sepehri Bonab et al(2024)	Flanker Task (measured by conflict cost scores)	Child	Objective (performance-based)
	Zhao et al(2022)	Psychoeducational Profile, Third Edition (PEP-3)-Cognitive	Clinician	Clinician-rated (semi-structured)
**Behavior problems**	de Vries et al(2015)	Disruptive Behavior Disorders Rating Scale(DBDRS)-ADHD	Parent	Subjective (proxy-report)
	Faja et al(2022)	Repetitive Behavior Scale-Revised (RBS-R)	Parent	Subjective (proxy-report)
	Fletcher-Watson et al(2016)	Brief observation of social communication change(BOSCC)	Independent trained examiner (video-coded parent–child interaction)	Objective (observational coding)
	Griffin et al(2021)	Social Skills Improvement System(SSIS)—problem behaviors	Parent	Subjective (proxy-report)
	Nekar et al(2022)	Repetitive Behavior Scale-Revised (RBS-R)-Stereotypic behavior	Parent	Subjective (proxy-report)
**Motor skills**	Soniyasri et al(2024)	Test of Gross Motor Development - Second Edition(TGMD-2)	Examiner (trained assessor)	Objective (standardized performance-based, examiner-rated)
	Vukićević et al(2019)	Developmental Assessment for Individuals With Severe Disabilities, second edition (DASH-2)	Examiner (special educator)	Objective (standardized performance-based, examiner-rated)
